# Unlocking surface octahedral tilt in two-dimensional Ruddlesden-Popper perovskites

**DOI:** 10.1038/s41467-021-27747-x

**Published:** 2022-01-10

**Authors:** Yan Shao, Wei Gao, Hejin Yan, Runlai Li, Ibrahim Abdelwahab, Xiao Chi, Lukas Rogée, Lyuchao Zhuang, Wei Fu, Shu Ping Lau, Siu Fung Yu, Yongqing Cai, Kian Ping Loh, Kai Leng

**Affiliations:** 1 Department of Chemistry, National University of Singapore, Singapore, Singapore; 2 Department of Applied Physics, The Hong Kong Polytechnic University, Hung Hom, Kowloon, Hong Kong, China; 3 Institute of Applied Physics and Materials Engineering, University of Macau, Macau, China

**Keywords:** Materials for optics, Surfaces, interfaces and thin films, Two-dimensional materials

## Abstract

Molecularly soft organic-inorganic hybrid perovskites are susceptible to dynamic instabilities of the lattice called octahedral tilt, which directly impacts their carrier transport and exciton-phonon coupling. Although the structural phase transitions associated with octahedral tilt has been extensively studied in 3D hybrid halide perovskites, its impact in hybrid 2D perovskites is not well understood. Here, we used scanning tunneling microscopy (STM) to directly visualize surface octahedral tilt in freshly exfoliated 2D Ruddlesden-Popper perovskites (RPPs) across the homologous series, whereby the steric hindrance imposed by long organic cations is unlocked by exfoliation. The experimentally determined octahedral tilts from *n* = 1 to *n* = 4 RPPs from STM images are found to agree very well with out-of-plane surface octahedral tilts predicted by density functional theory calculations. The surface-enhanced octahedral tilt is correlated to excitonic redshift observed in photoluminescence (PL), and it enhances inversion asymmetry normal to the direction of quantum well and promotes Rashba spin splitting for *n* > 1.

## Abstract (web summary)

The surface octahedral tilt in exfoliated 2D perovskites is directly visualized by STM and the degree of the tilt varies with the number of layers of inorganic slabs and result in different amounts of excitonic red shift in photoluminescence.

## Introduction

Organic-inorganic hybrid halide perovskites are prone to dynamic instabilities called octahedral tilt, which involves a rigid rotation of the inorganic octahedral cages, and can appear around any of the three Cartesian directions in the crystal with either in-phase or out-of-phase ordering^1,2^. Thermally induced octahedral tilt accompanies phase change in perovskites, and has a pronounced influence on electronic band structure including the band gap and band edge effective masses^3–8^. In conventional inorganic perovskites (with the general formula of ABX_3_, A is a monovalent cation, B is a divalent cation, and X is a halide/oxide anion), it is commonly accepted that the octahedral distortion is dominated by steric effect of the A site ion occupying the interstitial central cation site of BX_6_ octahedrons, in which the unmatched A and B ionic sizes induce octahedral tilt due to steric repulsion^9,10^. In hybrid organic-inorganic 3D perovskites, however, hydrogen bonding interactions between short organic A cation and the inorganic frame plays a central role in octahedral tilt^11–13^. For example, out-of-phase octahedra tilt occurs in the prototype MAPbI_3_ (MA = CH_3_NH_3_^+^) hybrid perovskite, while FAPbI_3_ (FA = (NH_2_)_2_CH^+^) hybrid perovskite shows almost no octahedral tilt due to the different hydrogen bonding interactions^14^. Octahedral tilt in 3D hybrid perovskites impacts the Jahn-Teller distortion of inorganic cages thus affecting the band gap^15,16^. In addition, the structural distortions are often coupled to vibrations, affecting the Raman mode frequencies and atomic motions related to the inorganic framework^17,18^. Octahedral tilt also induces exciton-phonon coupling and generates gap states which causes a broadening of the photoluminescence spectrum^19–25^.

Although Goldschmidt tolerance factors can semi-quantitatively predict structural distortion on the basis of steric hindrance in 3D perovskites, it is not straightforward to apply to 2D perovskites, because the layered segregated structures in 2D perovskites allow the incorporation of larger organic cations into the inorganic cages^26–30^. The apparent relaxation of Goldschmidt tolerance factor originates from the mitigation of strain accumulation and self-adjustable strain-balancing in structures of 2D perovskites. This has enabled a significantly expanded library of 2D RP lead halide perovskites with larger and more exotic A cations, leading to unique properties and novel applications^31–39^. Kepenakian et al. performed DFT calculations to understand how RPPs relieve mechanical strain at lattice-mismatched interfaces, and found that the internal elastic energy due to lattice strain is relieved by octahedral tilt of the PbX_6_ octahedra, which is more pronounced for RPPs above a certain critical thickness^40^.

X-ray diffraction (XRD) had been widely used to investigate octahedral tilt or lattice distortion in both 2D and 3D perovskites (see the summary of relevant works listed in Supplementary Table 1 and Table 2). However, XRD provides bulk averaged information with no surface sensitivity, thus it cannot be used to probe the presence of surface reconstruction that occurs on exfoliated RPPs. Here, we directly imaged octahedral tilts on the surface of 2D RPPs (BA)_2_(MA)_*n*−1_Pb_*n*_I_3*n*+1_ (*n* = 1–4; BA: CH_3_(CH_2_)_3_NH_3_, *n* refers to the number of inorganic slabs per unit cell of the homologous series) using scanning tunneling microscopy. We found that the octahedral tilt on the outermost layer of RPP was enhanced significantly compared to that of the bulk after “unlocking” the two interlocked layers of BA chains by mechanical exfoliation. In particular, the influence of MA ions on the octahedral tilt was investigated from *n* = 1 (without MA ions) to *n* = 4 (*n* > 1 has MA ions) RPPs, from which we identified a clear trend between *n* and the strength of the octahedral tilt. Interestingly, the out-of-plane octahedral tilt of the exfoliated surface is correlated to the redshifted emission peak alongside the primary exciton in the photoluminescence spectra, and also contributes to Rashba spin splitting.

## Results

### Unlocking surface octahedral tilt

**Fig. 1:**
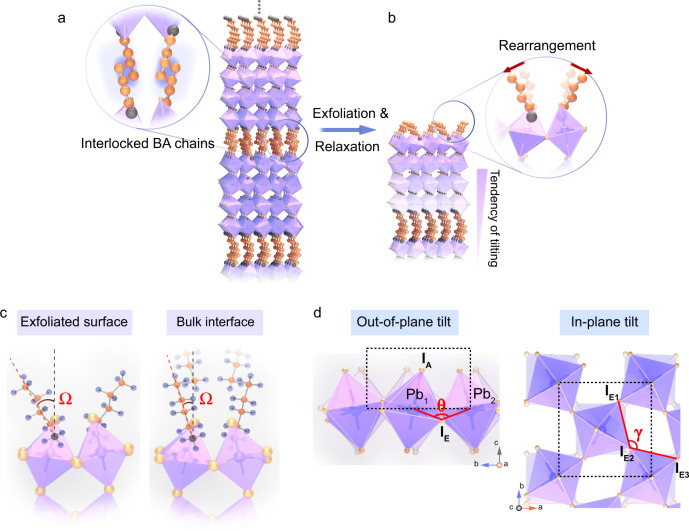
Structural analysis before and after unlocking the surface octahedral tilt. **a**, **b** Schematic illustration of exfoliation step liberating the steric constrain imposed by bilayer of BA cations in bulk and unlocking surface octahedral tilt in exfoliated RPP by taking *n* = 4 as an example. Two layers of BA chains are interlocked in the bulk (**a**) and leaving only one layer of BA on top upon splicing (**b**). **c** Simulation models of exfoliated monolayer surface and bulk interface. The tilt degree of BA molecule with respect to c axis is defined as *Ω* angle. **d** Schematic representation of the out-of-plane tilts and in-plane tilt of octahedral halide frame. *θ* and *γ* are used to measure the degree of octahedral tilt in out-of-plane and in-plane, respectively.

The mechanical exfoliation of van der Waals stacked 2D material (e.g., Graphene or transition metal dichalcogenide) usually does not induce large surface structural reorganization with respect to the bulk structure because of the lack of covalent bonds in the out-of-plane directions. However, the case is different for organic-inorganic hybrid 2D perovskites owing to its molecularly soft nature. As shown in Fig. 1a and b, delaminating one of the two interlocked layers of BA chains removes the steric constraints for the remaining, thus causing it to tilt at a larger angle relative to its bulk position. To gain an insight into the surface structure relaxation, we conducted DFT simulations on *n* = 4 RPP to model both exfoliated monolayer surface and bulk interface (Fig. 1c). The surface of relaxed monolayer structure has only one layer of BA on top, thus it is free from the steric constraints of interlocked BA bilayer and readily undergoes relaxation. The tilt angle (*Ω*) of the surface BA molecule with respect to the c-axis increases from 14.4° of the bulk, unrelaxed structure to 27.1° for the relaxed structure as illustrated in Table 1. This surface-enhanced octahedral tilt consists of a rigid rotation of the anion cage which can be specifically divided into the out-of-plane tilt (versus c-axis) and in-plane tilt (in ab plane). As shown in Fig. 1d, the Pb_1_–I_E_–Pb_2_ bond angle (*θ*) and I_E1_–I_E2_–I_E3_ bond angle (*γ*) are used to demonstrate out-of-plane and in-plane tilt, respectively. The degree of octahedral tilt is judged from the tilt angles of Δ*θ* (180°−*θ*) and Δ*γ* (ǀ90°−*γ*ǀ). In the out-of-plane tilt, the tilt of two octahedral cages toward each other narrows the *θ* angle by 5.2° from bulk interface to relaxed structure (Table 1). In the in-plane tilt, a stronger in-plane tilt degree is present in the monolayer surface (Δ*γ* = 3.2°) compared to the bulk interface (Δ*γ* = 1.6°). The corresponding bond length changes are summarized in Supplementary Figs. 1–4 and Tables 3–6.

**Table 1:** Comparison of the tilt angles of *n* = 4 RPP in monolayer surface and bulk interface according to DFT calculations.

		Exfoliated surface	Non-exfoliated surface
Tilt angle *Ω* (°) of BA		27.1	14.4
Surface octahedral tilt of *n* = 4 RPP	Pb_(1)_–I_E_–Pb_(2)_: *θ* (°)	145.3	150.5
	Out of plane tilt: Δ*θ* (°)	34.7	29.5
	I_E(1)_–I_E(2)_–I_E(3)_: *γ* (°)	93.2	88.4
	In-plane tilt: Δ*γ* (°)	3.2	1.6

### Atomic-scale STM measurement verifying DFT simulations

**Fig. 2:**
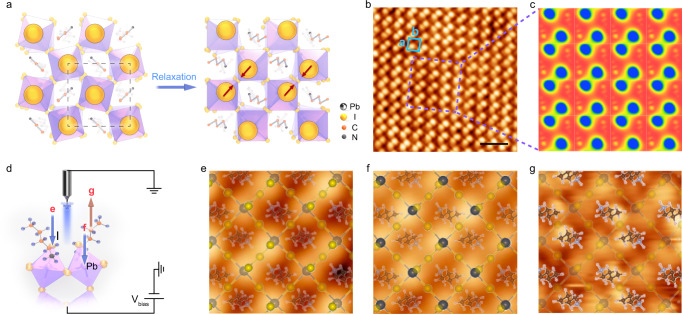
STM study of the octahedral tilt in exfoliated *n* = 4 RPP. **a** Schematic surface structure change by out-of-plane octahedral tilt in top view. The tilt leads to a “dimer-like” structure forming by two apical I atoms from the top of adjacent inorganic cages. **b** Atomic-resolution STM image of the exfoliated *n* = 4 RPP at *V*_bias_ = +2.3 V. The unit cell is marked as the blue rectangle with the vector **a** and **b**. Scale bar, 2 nm. **c** Simulated charge density plot. **d** Schematic of the directions of electron tunneling in STM measurement under different *V*_bias_. The different tunneling directions are marked by arrows. **e** STM image scanned at *V*_bias_ = +2.3 V showing apical I atoms. I atom is indicated as bright yellow sphere in the simulation model. **f** STM image scanned at *V*_bias_ = +1.9 V showing information of Pb atoms. Pb atom is indicated as black sphere in the simulation model. **g** STM image scanned at *V*_bias_ = −2.3 V showing BA molecules in the simulation model. The position of each atom or molecule in structural model fits well with STM result. STM images size of (**e**–**g**), 2 nm × 2 nm.

The visual surface structure of RPPs was imaged using STM measurement (see the detailed sample preparation process in the Supplementary Discussion and Supplementary Fig. 5). Large-sized, pure phase single crystals of (CH_3_(CH_2_)_3_NH_3_)_2_(CH_3_NH_3_)_*n*−1_Pb_*n*_I_3*n*+1_ of the homologous series *n* = 1–4 were synthesized, and thin flakes exfoliated from the bulk crystals were used for STM studies. According to the proposed model of exfoliated surface in monolayer *n* = 4 RPP, the stronger out-of-plane tilt (decreasing *θ* angle) of the two adjacent Pb–I cages toward each other will naturally bring their apical I atoms (large yellow spheres) closer, forming a “dimer-like” structure (Fig. 2a). This is indeed verified by STM real-space atomic-scale imaging of the surface structure of the exfoliated RPP flakes and cleaved CH_3_NH_3_PbI_3_ single crystals^41,42^. Figure 2b is a typical STM image of the *n* = 4 RPP on Au (111) substrate, showing a very flat surface with dimer-like structure representing two apical I atoms of the top inorganic Pb–I cages. The unit cell of the *n* = 4 RPP is marked by the blue rectangle, with lattice constant of **a** = 8.68 Å and **b** = 8.73 Å, fitting well with the (001) plane of the orthorhombic crystal. The large-scale STM and corresponding Fast Fourier Transition (FFT) images are shown in Supplementary Fig. 6, clearly verifying the orthogonal symmetry. Each unit cell contains two atoms (bright protrusions) that form a “dimer-like” structure, the distance of two nearest atoms is 4.58 Å, showing a very good agreement with the DFT relaxed model of monolayer structure of *n* = 4 (4.55 Å). The charge density plot is simulated as shown in Fig. 2c, most of the charge density is contributed by the terminal I atoms, thus the charge density plot reproduced the “dimer” structure under +2.3 V bias voltage.

The different orbitals of the 2D RPPs could be imaged by STM measurement by applying different bias voltages, as illustrated in Fig. 2d with the direction of electron tunneling marked by arrows. The energies of different atomic orbitals are different with respect to the Fermi level, thus they are accessed at different bias voltages (see the calculated local density of states in Supplementary Fig. 7). When applied positive bias is larger than +2.0 V (electrons tunneling from tip to sample), apical I atoms are imaged (Fig. 2e). Reducing the voltage to +1.9 V allows the Pb atoms of *n* = 4 RPP to be imaged (Fig. 2f), where a nearly square lattice with dimensions of 5.89 Å was observed, consistent with the Pb–Pb bond length in the calculation model (6.09 Å). A negative bias of −2.3 V (electrons tunneling from sample to tip) is required to image the organic BA cations, as shown in Fig. 2g. Applying bias during the STM measurement did not distort the lattice, this has been verified by bias-variable STM measurements and STM simulations shown in the Supplementary Figs. 8 and 9. The experimental STM images are consistent with simulated STM images at different bias voltages. The different topologies of STM images at +1.9 V and +2.3 V are attributed to contributions from Pb and I orbitals, respectively.

### Layer-dependent octahedral tilt

#### DFT studies

**Fig. 3:**
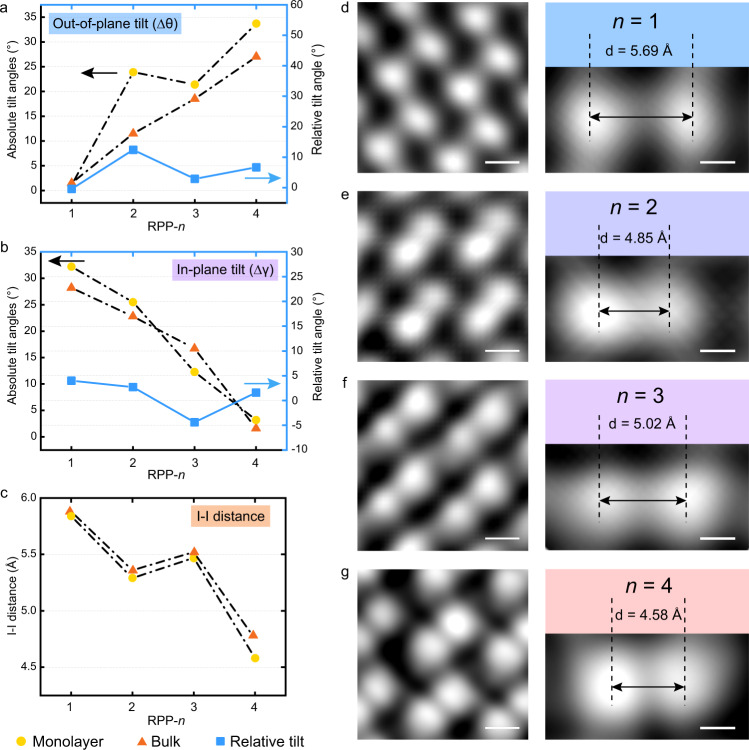
The degree of octahedral tilt as a function of dimensionality *n* in RPPs. **a**, **b** Plot summarizing out-of-plane and in-plane octahedral tilt in the monolayer surface (yellow circles) and bulk interface (orange triangles) of *n* = 1, 2, 3, 4 RPP series according to the simulation models. The relative tilt (blue rectangles) is defined as the difference in tilt angle between monolayer and bulk octahedral tilt angle. **c** The distance of two apical I atoms from the top of octahedrons according to the simulation models. **d**–**g** Enlarged view of STM images scanned on freshly exfoliated RPP surfaces of *n* = 1–4. The measured layer-dependent apical I–I distance fits well with the simulation results. Scale bars, 0.4 nm (left part of **d**–**g**); 0.2 nm (right part of **d**–**g**). STM setpoints: *V*_bias_ = +2.3 V, *I* = 30 pA.

To investigate the surface octahedral tilt as a function of *n* in 2D RPPs, DFT calculations were carried out on both monolayer and bulk RPPs from *n* = 1 to *n* = 4. The simulated structures include fully relaxed ground state (GS) of orthorhombic structures, and a “high symmetry” (HS) counterpart where all octahedrons are fixed and un-tilted (Supplementary Fig. 10). The degree of absolute octahedral tilt is determined from the tilt angles (Δ*θ*_n_ and Δ*γ*_n_, *n* is the thickness of octahedron) with respect to the reference HS structure, the result is summarized in Fig. 3a and b (detailed bond angle and bond length are shown in Supplementary Figs. 1–4 and Tables 3–6). Among *n* = 1 to *n* = 4, monolayer *n* = 4 RPP shows the strongest surface out-of-plane octahedral tilt with a tilt degree Δ*θ*_4_ = 34.7°, while it has the smallest in-plane tilt with Δ*γ*_4_ = 3.2°. In contrast, the *n* = 1 homolog shows almost no out-of-plane tilt but the largest absolute in-plane tilt of Δ*γ*_1_ = 32.2°. The apical I–I distance (*L*_n_) between the adjacent surface PbI_6_ cages varies from *n* = 1 to 4 in monolayer surface as shown in Fig. 3c, with the shortest I–I distance in *n* = 4 and the longest in *n* = 1 RPP. Interestingly, the apical I–I distance in *n* = 2 is slightly shorter than that in *n* = 3, thus the I–I distance does not change monotonically, but depends on the interplay of various Coulombic interactions with other ions (e.g., methylammonium ions) in the structure. We should note that besides the absolute tilt angle measured with respect to a non-tilted imaginary reference HS structure, it is more important to look at the relative tilt angle (marked as blue rectangles in Fig. 3a and b) because in the relaxed bulk system, it already has an intrinsic octahedral tilt relative to un-tilted HS reference. Here, the relative tilt angle denotes the difference value between exfoliated monolayer and non-exfoliated bulk system. In the following section, we correlate the experimentally observed trends to the relative tilt.

#### STM

The experimental atomic arrangements of the apical I atoms from *n* = 1 to *n* = 4 (Fig. 3d–g) exfoliated RPPs were imaged by STM. All the bright protrusions are due to apical I atoms from *n* = 1 to *n* = 4 RPPs, which has been verified by the DFT calculation of LDOS and corresponding STM image simulations (Supplementary Figs. 11 and 12). To our delight, the STM results verified the DFT-predicted trend of apical I–I distance of monolayer RPP structures from *n* = 1 to *n* = 4. STM shows that *n* = 4 RPP (Fig. 3g) has the shortest apical I–I distance of 4.55 Å, while *n* = 2 RPP has shorter I–I distance of 4.85 Å (Fig. 3e) than *n* = 3 RPP of 5.02 Å (Fig. 3f). Meanwhile, *n* = 1 RPP (Fig. 3d) has the largest I–I distance of 5.69 Å, which fits well with the simulated model that almost no out-of-plane tilt occurred in the surface octahedron of *n* = 1 RPP. These results clearly validate that the out-of-plane surface octahedral tilt is directly correlated to the I–I distance, while in-plane octahedral tilt has less correlations with it.

### Excitonic redshift in the exfoliated RPP crystals

**Fig. 4:**
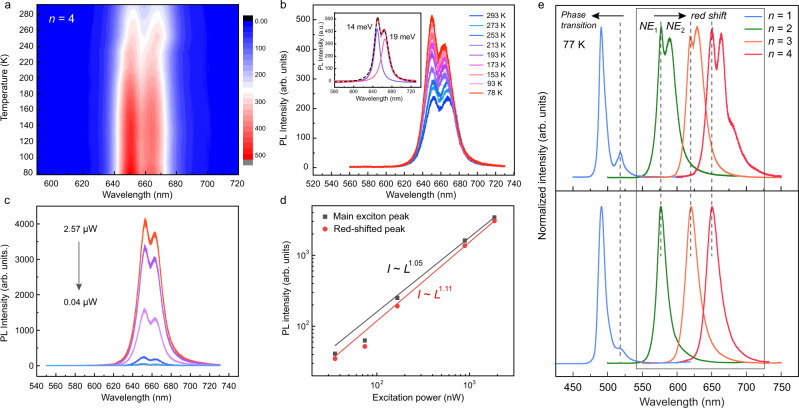
Layer-dependent excitonic emissions in exfoliated RPP flakes from *n* = 1 to *n* = 4. **a** Photoluminescence (PL) color map showing two excitonic emissions in surface exfoliated *n* = 4 RPP as different temperatures. **b** Temperature-dependent PL measurement in *n* = 4 RPP. Inset, a typical redshifted narrow emission peak with full width at half maximum (FWHM) of 19 meV in addition to the primary exciton with FWHM of 14 meV. **c** Power-dependent PL measurement in *n* = 4 RPP. **d** PL integrated intensity of the two excitonic emissions in *n* = 4 RPP as a function of laser power. **e** PL measurements of the exfoliated (up) and non-exfoliated bulk crystals (down) from *n* = 1 to *n* = 4 RPPs at 77 K. Redshifted PL peak appears for *n* ≥ 2 RPPs, whereas *n* = 1 RPP shows blue-shifted PL peak caused by phase transition instead.

A single narrow photoluminescence (PL) peak due to the recombination of free exciton characterizes the emission of single-crystalline RPP crystals of *n* = 1 to 4 homologs series^43^. However, the PL spectrum of exfoliated RPP flakes of ~100–150 nm thickness (Supplementary Fig. 13) reveals a sharp redshifted emission peak in addition to the main exciton peak, as shown in Fig. 4a, which is distinct from defect-related broad emissions (BE)^44^. Using *n* = 4 RPP as an example, the redshifted narrow emission peak (NE2, full width at half maximum, FWHM ~19 meV, Fig. 4b inset) at 665 nm appears just alongside the main exciton peak (NE1, FWHM ~14 meV, Fig. 4b inset). Temperature-dependent PL of *n* = 4 (Fig. 4b) shows that peak NE2 becomes slightly weaker in relation to the primary exciton peak NE1 upon temperature reduction to 77 K. Power-dependent PL measurement (Fig. 4c) verifies the excitonic nature of the redshifted emission peaks, where the integrated PL intensity can be fitted by the power-law relation with respect to the excitation intensity *L* as *I* ~ *L*^*k*^ with *k*_NE1_ = 1.05 and *k*_NE2_ = 1.11 (Fig. 4d). Similar data for *n* = 2 and *n* = 3 are shown in Supplementary Fig. 14 and Fig. 15. Furthermore, the energy shifts between the two exciton peaks were analyzed across the RPP homologs series as shown in Fig. 4e. Here, *n* = 2 RPP flake shows the largest shift with Δ*E*_2_ = 44.7 meV, followed by *n* = 4 (Δ*E*_4_ = 37.4 meV) and *n* = 3 (Δ*E*_3_ = 30.4 meV) RPPs. This trend matches the relative out-of-plane octahedral tilt angle (blue rectangles in Fig. 3a) across the homologous series. In contrast to *n* > 1, the exciton peak blue-shifts in *n* = 1 RPP due to phase transition of the organic cations at low temperatures^18^, and it seems that phase shift is allowed in *n* = 1 due to the very weak out-of-plane octahedral tilt. Importantly, the redshifted emission is uniform across the surface and is not related to edge emission^45,46^. It also does not originate from thickness-dependent photon recycling because covering the surface with hexagonal boron nitride (h-BN) removes the redshifted PL, independent of the flake thickness.

**Fig. 5:**
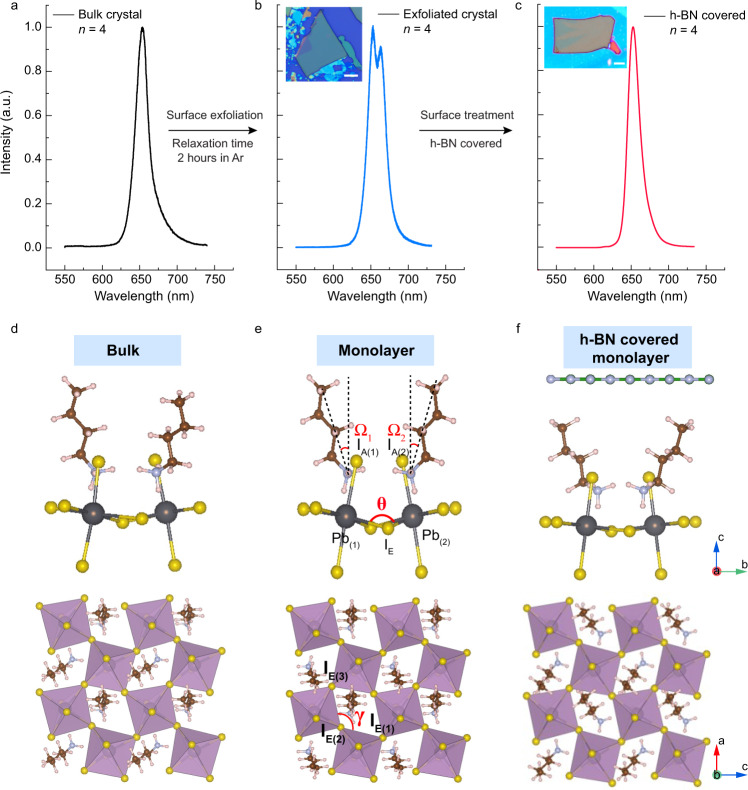
Excitonic redshift in the exfoliated RPP crystals is suppressed by h-BN covering. **a** Single PL emission in *n* = 4 RPP single crystal. **b** PL emission with a redshifted peak next to the primary peak for exfoliated bulk *n* = 4 RPP; inset, optical image of a typical exfoliated *n* = 4 RPP flake. Scale bar, 10 μm. **c** Absence of redshifted PL emission by encapsulating freshly exfoliated *n* = 4 RPP flake with h-BN. Inset, Optical image of a typical h-BN covered *n* = 4 RPP flake. Scale bar, 20 μm. **d**–**f** Optimized geometry of *n* = 2 RPP of **d** bulk crystal; **e** monolayer and **f** h-BN covered monolayer. Δ*θ* and Δ*γ* are representative out-of-plane and in-plane tilt angles, respectively.

Since the out-of-plane octahedral tilt occurs after delamination, it is instructive to ask if the tilt can be suppressed if a monolayer of another material was stacked on top by van der Waals interaction. In view of the fact that h-BN has a large band gap (~6 eV) and is optically transparent and inert, we used it as a top confinement layer on freshly exfoliated RPP to examine whether it can suppress surface reconstruction and prevent the redshifted emission. Strikingly, the redshifted emission (Fig. 5a and b) was only observed for freshly exfoliated RPPs after allowing the surface to relax in an inert atmosphere. If the exfoliated sample was immediately covered with h-BN, the redshifted emission was suppressed (Fig. 5c). To generate similar redshift in the PL for the h-BN confined sample, additional energy input in the form of laser annealing is needed as reported previously^39^. To understand the interfacial interaction, the h-BN/RPP heterostructure was modeled using DFT by stacking a monolayer of h-BN on *n* = 2 RPP monolayer and allowing the heterostructure to relax. As illustrated in Fig. 5d–f, we compare the changes in bond angles (out-of-plane tilt: Δ*θ* and in-plane tilt: Δ*γ*) and bond lengths (Supplementary Table 7) between the *n* = 2 bulk structure, monolayer, and h-BN covered monolayer. According to our simulations, the h-BN covered monolayer *n* = 2 RPP shows a very similar out-of-plane tilt angle (12.4°) as the bulk *n* = 2 RPP (11.5°), while it has a larger in-plane tilt (27.5°) than the bulk crystal (Table 2). Therefore, DFT simulation verifies that the out-of-plane relaxation is suppressed in the presence of h-BN confinement layer compared with the uncovered monolayer perovskites.

**Table 2:** Comparison of the tilt angle of *n* = 2 RPP in non-exfoliated bulk interface, monolayer surface, and h-BN covered monolayer surface according to the simulation models.

		Bulk	Monolayer	h-BN covered monolayer
Bond angle of *n* = 2 RPP	BA tilt angle *Ω*_1_; *Ω*_2_ (°), respectively	13.3; 16.1	19.5; 17.5	26.9; 26.6
	Out of plane tilt: Δ*θ* (°)	11.5	24.7	12.4
	In-plane tilt: Δ*γ* (°)	22.8	25.5	27.5

One possibility for the redshifted PL is the coupling of the excitons to optical phonon arising from torsional motion of BA chains^22,23,47,48^; the corresponding exciton-phonon Fröhlich coupling strength was reported to be around 30 to 60 meV, which agrees well with the redshifted PL of several tens of meV (e.g., Δ*E*_2_ = 44.7 meV). Besides, our temperature-dependent Raman measurement is performed in exfoliated *n* = 2 RPP. As shown in Supplementary Fig. 16, the vibrational modes around 25 and 250 cm^−1^ are associated with torsional mode of the NH_3_^+^ head of the organic spaces that is coupled to excitonic transitions^48,49^. On the other hand, our DFT simulation based on both nonadiabatic molecular dynamic and time-dependent DFT (TDDFT) methods corroborates the presence of a redshifted exitonic peak in exfoliated *n* = 2 RPP in addition to the main exciton peak seen in the bulk crystal (Supplementary Fig. 17).

## Discussion

The results collected above allow us to examine the microscopic mechanism of surface octahedral tilt and its dependence on the *n*th dimensionality of 2D hybrid perovskites. According to our DFT calculations, octahedral tilt is intrinsic to the PbI_6_ octahedral cage and occurs in bulk 2D RPPs. Depending on the interplay of electrostatic, steric, and hydrogen bonding interactions of BA^+^ and MA^+^ cations, the degree of octahedral tilt varies from *n* = 1 to *n* = 4 RPPs. Our finding is that in the exfoliated structure, the surface octahedral tilt is even stronger than the bulk and detailed structure comparisons of these two are demonstrated in Supplementary Fig. 18 and Tables 8–10. Similar to 3D hybrid perovskites, MA cations play a vital role in tuning the bulk octahedral tilt: (1) in *n* = 1 RPP where MA cations are absent, only in-plane tilt occurs; (2) in *n* ≥ 2 RPPs, absolute out-of-plane octahedral tilt increases due to steric effects from MA molecules, shown as orange triangles in the Fig. 3a. The out-of-plane octahedral tilt is stronger in *n* > 1 RPPs homologues that have a greater number of inorganic layers, this is due to the accumulating internal charge and structural mismatch with added MA ions.

**Fig. 6:**
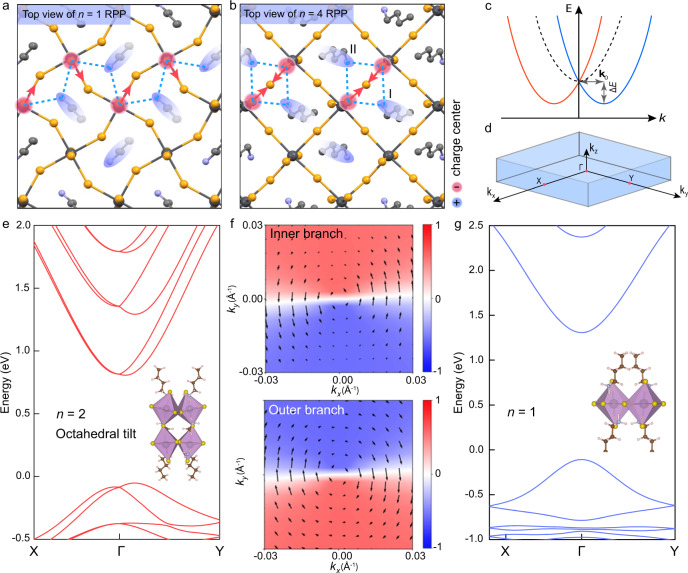
Rashba band splitting shown in exfoliated RPPs of *n* > 1. **a**, **b** Varying configurations of the top BA molecule and N–H···I hydrogen bond between BA and inorganic cage in surface of monolayer *n* = 1 (**a**) and *n* = 4 (**b**). **c** Schematic band diagram showing the bands shifted in *k*-space by ***k***_0_. An indirect band gap is displayed with an energy Δ*E* below the direct band gap. **d** High symmetry points of the Brillouin zone in *n* = 2 RPP structure. **e** Dispersed band structures of monolayer *n* = 2 RPP along X–Γ–Y directions, showing the Rashba band splitting feature. Inset, asymmetric structure of monolayer *n* = 2 RPP with enhanced surface octahedral tilt. **f** Spin-textures of valance bands projected on the 2D X–Y plane near Γ point, where the inner and outer bands have anti-clockwise and clockwise in-plane spin-textures, respectively. The color code represents the *S*_z_ spin component. **g** Simulated band structure of monolayer *n* = 1 RPP along X–Γ–Y direction showing no Rashba splitting. Inset, centrosymmetric structure of monolayer *n* = 1 RPP without obvious octahedral tilt.

Interestingly, the surface octahedral tilt generates varying configurations of the top BA molecules owing to the minimization of electrostatic energies and steric repulsions. As shown in Fig. 6a and b, the BA organic chain is a polar molecule with a positively charged “head” of NH_3_^+^ ion (shaded as blue) and a negatively charged “tail” of C_4_H_9_^−^ ion (shaded as white). We compare the DFT-optimized surface structures of monolayer *n* = 1 (Fig. 6a) and *n* = 4 (Fig. 6b) RPPs to reveal how the configuration of BA molecules is commensurate with the octahedral tilt on account of the N–H···I hydrogen bond connecting them. In *n* = 1 RPP, the BA cations interlaced with each other due to the interaction with apical I atom (shaded as red) of surface PbI_6_ octahedron, where a larger in-plane octahedral tilt and a small out-of-plane tilt exist. In the case of *n* = 4 RPP, the BA molecules adopt a “head-to-head” configuration so that their NH_3_ groups are positioned to have strong hydrogen bonding interactions with the two apical I atoms of surface PbI_6_ octahedron with large out-of-plane tilt, resulting in alternating long and short I–I distances at surface that could be seen as the apparent “dimer” structures in the STM image of Fig. 3g.

We compare the surface (interface) octahedral tilt between monolayer and bulk structures according to the DFT simulations. The out-of-plane octahedral tilt was enhanced in exfoliated *n* ≥ 2 RPPs. *n* = 2 has the largest relative tilt angle of 13.9° (Δ*θ*_Exfoliated_ − Δ*θ*_Bulk_), following by *n* = 4 (5.2°) and *n* = 3 RPPs (3.1°), as shown by the blue rectangle plot in Fig. 3a. This trend is consistent with the energy shift between the two exciton peaks in the PL measurements (Fig. 4e), where *n* = 2 has the largest shift. On the other hand, the in-plane tilt is less correlated with the redshifted exciton peaks observed in PL. One possible explanation is that the out-of-plane octahedral tilt results in a stronger vibronic coupling of the organic cations with the lead iodide octahedral cage compared to the in-plane octahedral tilt.

The structural relaxation at the top layers is transmitted to the subsurface layers due to the hydrogen bonding interactions that causes synergistic movement of PbI_6_ cages. Although the structural distortion is expected to decrease with distances from the surface, it must also necessarily imply that structural asymmetry builds up in the direction perpendicular to the plane of the quantum well (Supplementary Figs. 19–21 and Tables 11–13). The inversion asymmetric potential may lift the spin degeneracy of two-dimensional (2D) electronic bands—the so-called Rashba effect, causing it to split into two spin-polarized bands (Fig. 6c), where the momentum offset (**k**_0_) refers to the distance between the extrema of the splitting band and the high symmetry point in k-space, and the splitting energy (Δ*E*) is the energy difference between them. Here, the Rashba splitting parameter is defined as: ${\alpha }_{{{{{{\rm{R}}}}}}}=2\Delta E/{{{{{{\bf{k}}}}}}}_{0}$.

DFT calculations were performed to see if the strength of the Rashba band splitting is correlated to the octahedral tilt observed for the RPP crystal^50^. The Brillouin zone and corresponding Rashba spin-polarized bands of the monolayer *n* = 2 structure are shown in Fig. 6d and e, in which the band minimum of the split band shifts from the high symmetry Γ point along X–Γ–Y directions. The band splitting energy and Rashba splitting coefficients are Δ*E* = 9 meV, *α*_R_ = 1.15 eV·Å for electron and Δ*E* = 34 meV, *α*_R_ = 1.55 eV·Å for hole along the Γ–Y band direction. The indirect band gap is 42 meV lower than the direct band gap at Γ point. The corresponding Rashba-type spin-textures of the spin-split valance bands are projected onto the 2D X–Y plane near Γ point in Fig. 6f. In the inner and outer branches, the sense of rotation (anti-clockwise and clockwise) of the spin-textures are opposite due to the spin–orbit interaction. By contrast, the band structure of the monolayer *n* = 1 RPP (Fig. 6g) shows no spin splitting. In view of the fact, there is minimal out-of-plane octahedral tilt in *n* = 1 as compared to strong out-of-plane octahedral tilt in *n* = 2, we can infer that the strength of the Rashba band splitting may be correlated to the out-of-plane octahedral tilt, among other factors.

Our studies show that the surface pairing of iodine atoms observed in the STM images of exfoliated 2D RPP perovskites is correlated to the out-of-plane octahedral tilt of the Pb–I cage at the surface, which is accentuated compared with the bulk. The octahedral tilt is a result of balancing steric forces and hydrogen bonding interactions with methylammonium ions. The surface-enhanced out-of-plane octahedral tilt is correlated to the emergence of a redshifted peak in the photoluminescence spectra, where the extent of the redshifts can be correlated to the relative octahedral tilt (surface versus bulk) from *n* = 2 to *n* = 4. Nonadiabatic molecular dynamic and TDDFT methods suggest that as a result of the surface octahedral tilt, sub-gap excitonic states are created and these can give rise to the redshifted PL. In addition, out-of-plane octahedral tilt at the surface enhances inversion asymmetry, and DFT calculations show that Rashba spin splitting is pronounced for *n* > 1 RPP perovskites that exhibit enhanced out-of-plate octahedral tilt.

## Methods

2D RPP single crystals with the chemical formula (BA)_2_(MA)_*n*−1_Pb_*n*_I_3*n*+1_ (*n* = 1–4) were synthesized via a temperature-programmed solution precipitation method which have been reported before^39^. The reaction stoichiometry of three precursors of PbO, BAI and MAI are well controlled in a concentrated HI and H_3_PO_2_ to guarantee the phase pure of the homologs. Here, taking (BA)_2_(MA)_3_Pb_4_I_13_ *n* = 4 as an example, PbO (0.69 M), BAI (0.17 M), and MAI (0.52 M) precursors were dispersed in a concentrated HI and H_3_PO_2_ mixture (7.6:1, vol/vol), and then heated to 110 °C with stirring until a clear solution is obtained. The solution was quickly transferred to an oven at 110 °C and allowed to cool slowly to room temperature at a rate of 3 °C h^−1^, whereupon metallic black square-shaped crystals started to form. Finally, the obtained single crystals were dried in an Ar-filled vacuum chamber at room temperature.

The freshly prepared 2D RPP bulk crystals are exfoliated on Si/SiO_2_ substate and Au (111) substrate in Ar-filled glove box by mechanical exfoliation method and transferred into HV and UHV chambers for optical and STM measurements, respectively. During the transfer process, the samples are kept in inert atmosphere preventing decomposition. For h-BN covered RPPs, A few-layers h-BN was exfoliated first before covering on freshly exfoliated RPP crystals by dry transfer technology in glove box.

### STM measurements

STM measurements were carried out in ultrahigh vacuum with a base pressure of 2 × 10^−11^ mbar using a commercial Omicron LT STM system. All STM images were collected under constant-current mode with electro-chemically etched tungsten tips and all given voltages refer to the sample. Before STM measurements, the tip was conditioned and calibrated on a clean Au (111) surface. Images were collected at 4.5 K.

### Steady-state photoluminescence measurement

The 400-nm fs laser pulses, which were obtained by frequency doubling of the 800-nm fs laser pulses (50 fs, 1 kHz) generated from a Ti: sapphire laser (Coherent Libra-HE) via the use of a BBO crystal, were used as the excitation light for the PL measurement. The 400-nm fs laser beam was focused onto the sample by an objective lens (Olympus LMPLFLN 50X/0.5). The PL emission from the surface of the sample was collected by the same objective lens and then coupled to a spectrum analyzer (Princeton SpectraPro 2750 integrated with a Princeton EMCCD) for spectrum analysis. For low-temperature (i.e., from 77 to 350 K) PL measurement, the sample was kept inside a vacuum chamber (Linkam optical DSC600 temperature-controlled stage) with an optical window. The optical window, which was purged with N_2_ gas, was used to perform PL measurement at low temperature.

### First-principles simulation

Density functional theory (DFT) calculations were performed with the Vienna ab initio simulations package (VASP)^51^. The Perdew-Burke-Ernzerhof (PBE) type of the generalized gradient approximation (GGA) was used as the exchange-correlation functional^52^. The van der Walls interactions were described by the DFT-D2 method^53^. A plane wave cutoff energy of 400 eV with a 3 × 3 × 1 Monkhorst–Pack grid was adopted for structure optimization, whereas a denser 5 × 5 × 1 grid for electronic calculation. All the structures were completely relaxed until the force on the atoms is <0.01 eV/Å. The (BA)_2_(MA)_*n*−1_Pb_*n*_I_3*n*+1_ (010) surface was represented using a periodic slab based on the space group Cc2m containing *n* inorganic atomic layers with a vacuum thickness of 15 Å. The spin-orbital coupling was considered for the Rashba band and spin texture calculation.

## Supplementary information

Supplementary Information

Peer Review File

## Data Availability

All data generated and analyzed in this study are included in the Article and its Supplementary Information, and are also available from authors upon request.

## References

[1] Glazer A (1972). Classification of tilted octahedra in perovskites. Acta Crystallogr., Sect. B: Struct. Crystallogr. Cryst. Chem..

[2] Mitzi DB (1999). Synthesis, structure and properties of organic-inorganic perovskites and related materials. Prog. Inorg. Chem..

[3] Stoumpos CC, Malliakas CD, Kanatzidis MG (2013). Semiconducting tin and lead iodide perovskites with organic cations: phase transitions, high mobilities, and near-infrared photoluminescent properties. Inorg. Chem..

[4] Angel RJ, Zhao J, Ross NL (2005). General rules for predicting phase transitions in perovskites due to octahedral tilting. Phys. Rev. Lett..

[5] Wu B (2019). Indirect tail states formation by thermal-induced polar fluctuations in halide perovskites. Nat. Commun..

[6] Prasanna R (2017). Band gap tuning via lattice contraction and octahedral tilting in perovskite materials for photovoltaics. J. Am. Chem. Soc..

[7] Lufaso MW, Woodward PM (2004). Jahn-Teller distortions, cation ordering and octahedral tilting in perovskites. Acta Crystallogr., Sect. B.

[8] Lanigan-Atkins T (2021). Two-dimensional overdamped fluctuations of the soft perovskite lattice in CsPbBr_3_. Nat. Mater..

[9] Filip MR, Eperon GE, Snaith HJ, Giustino F (2014). Steric engineering of metal-halide perovskites with tunable optical band gaps. Nat. Commun..

[10] Motta C (2015). Revealing the role of organic cations in hybrid halide perovskite CH_3_NH_3_PbI_3_. Nat. Commun..

[11] Lee J-H (2016). Resolving the physical origin of octahedral tilting in halide perovskites. Chem. Mater..

[12] Lee J-H, Bristowe NC, Bristowe PD, Cheetham AK (2015). Role of hydrogen-bonding and its interplay with octahedral tilting in CH_3_NH_3_PbI_3_. Chem. Commun..

[13] Varadwaj PR, Varadwaj A, Marques HM, Yamashita K (2019). Significance of hydrogen bonding and other noncovalent interactions in determining octahedral tilting in the CH_3_NH_3_PbI_3_ hybrid organic-inorganic halide perovskite solar cell semiconductor. Sci. Rep..

[14] Amat A (2014). Cation-induced band-gap tuning in organohalide perovskites: interplay of spin-orbit coupling and octahedra tilting. Nano Lett..

[15] Varignon J, Bibes M, Zunger A (2019). Origin of band gaps in 3d perovskite oxides. Nat. Commun..

[16] Neukirch AJ (2018). Geometry distortion and small polaron binding energy changes with ionic substitution in halide perovskites. J. Phys. Chem. Lett..

[17] Wei T-C (2017). Photostriction of CH_3_NH_3_PbBr_3_ perovskite crystals. Adv. Mater..

[18] Menahem M (2021). Strongly anharmonic octahedral tilting in two-dimensional hybrid halide perovskites. ACS Nano.

[19] Cortecchia D (2017). Broadband emission in two-dimensional hybrid perovskites: the role of structural deformation. J. Am. Chem. Soc..

[20] Luo J (2018). Efficient and stable emission of warm-white light from lead-free halide double perovskites. Nature.

[21] Iaru CM, Geuchies JJ, Koenraad PM, Vanmaekelbergh D, Silov AY (2017). Strong carrier-phonon coupling in lead halide perovskite nanocrystals. ACS Nano.

[22] Wright AD (2016). Electron-phonon coupling in hybrid lead halide perovskites. Nat. Commun..

[23] Straus DB (2016). Direct observation of electron-phonon coupling and slow vibrational relaxation in organic-inorganic hybrid perovskites. J. Am. Chem. Soc..

[24] Baldwin A (2021). Local energy landscape drives long-range exciton diffusion in two-dimensional halide perovskite semiconductors. J. Phys. Chem. Lett..

[25] Gong X (2018). Electron-phonon interaction in efficient perovskite blue emitters. Nat. Mater..

[26] Stoumpos CC (2016). Ruddlesden–Popper hybrid lead iodide perovskite 2D homologous semiconductors. Chem. Mater..

[27] Grancini G, Nazeeruddin MK (2018). Dimensional tailoring of hybrid perovskites for photovoltaics. Nat. Rev. Mater..

[28] Smith, M. D., Crace, E. J., Jaffe, A. & Karunadasa, H. I. The diversity of layered halide perovskites. *Annu. Rev. Mater. Res*. **48**, 111–136 (2018).

[29] Saparov B, Mitzi DB (2016). Organic–inorganic perovskites: structural versatility for functional materials design. Chem. Rev..

[30] Soe CMM (2017). New type of 2D perovskites with alternating cations in the interlayer space, (C(NH_2_)_3_)(CH_3_NH_3_)_n_Pb_n_I_3n+1_: structure, properties, and photovoltaic performance. J. Am. Chem. Soc..

[31] Tsai H (2016). High-efficiency two-dimensional Ruddlesden-Popper perovskite solar cells. Nature.

[32] Li L (2019). Two-dimensional hybrid perovskite-type ferroelectric for highly polarization-sensitive shortwave photodetection. J. Am. Chem. Soc..

[33] Zhai Y (2017). Giant Rashba splitting in 2D organic-inorganic halide perovskites measured by transient spectroscopies. Sci. Adv..

[34] Leng K, Li R, Lau SP, Loh KP (2021). Ferroelectricity and Rashba effect in 2D organic–inorganic hybrid perovskites. Trends Chem..

[35] Xiao X (2021). Layer number dependent ferroelasticity in 2D Ruddlesden-Popper organic-inorganic hybrid perovskites. Nat. Commun..

[36] Leng K, Fu W, Liu Y, Chhowalla M, Loh KP (2020). From bulk to molecularly thin hybrid perovskites. Nat. Rev. Mater..

[37] Park IH (2019). Ferroelectricity and Rashba effect in a two-dimensional Dion-Jacobson hybrid organic-inorganic perovskite. J. Am. Chem. Soc..

[38] Leng K (2020). Electron tunneling at the molecularly thin 2D perovskite and graphene van der Waals interface. Nat. Commun..

[39] Leng K (2018). Molecularly thin two-dimensional hybrid perovskites with tunable optoelectronic properties due to reversible surface relaxation. Nat. Mater..

[40] Kepenekian M (2018). Concept of lattice mismatch and emergence of surface states in two-dimensional hybrid perovskite quantum wells. Nano Lett..

[41] She L, Liu M, Zhong D (2016). Atomic structures of CH_3_NH_3_PbI_3_ (001) surfaces. ACS Nano.

[42] Ohmann R (2015). Real-space imaging of the atomic structure of organic–inorganic perovskite. J. Am. Chem. Soc..

[43] Ishihara T, Takahashi J, Goto T (1990). Optical properties due to electronic transitions in two-dimensional semiconductors (C_n_H_2n+1_NH_3_)_2_PbI_4_. Phys. Rev. B.

[44] Kahmann S, Tekelenburg EK, Duim H, Kamminga ME, Loi MA (2020). Extrinsic nature of the broad photoluminescence in lead iodide-based Ruddlesden–Popper perovskites. Nat. Commun..

[45] Blancon J-C (2017). Extremely efficient internal exciton dissociation through edge states in layered 2D perovskites. Science.

[46] Shi E (2019). Extrinsic and dynamic edge states of two-dimensional lead halide perovskites. ACS Nano.

[47] Ni L (2017). Real-time observation of exciton-phonon coupling dynamics in self-assembled hybrid perovskite quantum wells. ACS Nano.

[48] Urban JM (2020). Revealing excitonic phonon coupling in (PEA)_2_(MA)_n-1_Pb_n_I_3n+1_ 2D layered perovskites. J. Phys. Chem. Lett..

[49] Dhanabalan B (2020). Directional anisotropy of the vibrational modes in 2D-layered perovskites. ACS Nano.

[50] Marronnier A (2018). Influence of disorder and anharmonic fluctuations on the dynamical Rashba effect in purely inorganic lead-halide perovskites. J. Phys. Chem. C..

[51] Kresse G, Furthmüller J (1996). Efficient iterative schemes for ab initio total-energy calculations using a plane-wave basis set. Phys. Rev. B.

[52] Perdew JP, Burke K, Ernzerhof M (1996). Generalized gradient approximation made simple. Phys. Rev. Lett..

[53] Grimme S (2006). Semiempirical GGA-type density functional constructed with a long-range dispersion correction. J. Comput. Chem..

