# Supplementary material for: Unlocking surface octahedral tilt in two-dimensional Ruddlesden-Popper perovskites
Source: Nat Commun. 2022 Jan 10;13:138. doi: 10.1038/s41467-021-27747-x (PMC8748742; doi:10.1038/s41467-021-27747-x)
Supplement: Supplementary file 1 — Supplementary Information [file 41467_2021_27747_MOESM1_ESM.pdf]

Supplementary Information

Unlocking surface octahedral tilt in two-dimensional Ruddlesden-Popper perovskites

Yan Shao[1#], Wei Gao[2#], Hejin Yan[3], Runlai Li[1], Ibrahim Abdelwahab[1], Xiao Chi[1], Lukas Rogée[2], Lyuchao Zhuang[2], Wei Fu[1], Shu Ping Lau[2], Siu Fung Yu[2]*, Yongqing Cai[3]*, Kian Ping Loh[1]* and Kai Leng[2]*

[1]Department of Chemistry, National University of Singapore, Singapore, Singapore

[2]Deapartment of Applied Physics, The Hong Kong Polytechnic University, Hung Hom, Kowloon, Hong Kong, China

[3]Institute of Applied Physics and Materials Engineering, University of Macau, Macau, China

[#]These authors contributed equally: Yan Shao, Wei Gao

*e-mail: siu.fung.yu@polyu.edu.hk; yongqingcai@um.edu.mo; chmlohkp@nus.edu.sg; kathy-kai.leng@polyu.edu.hk

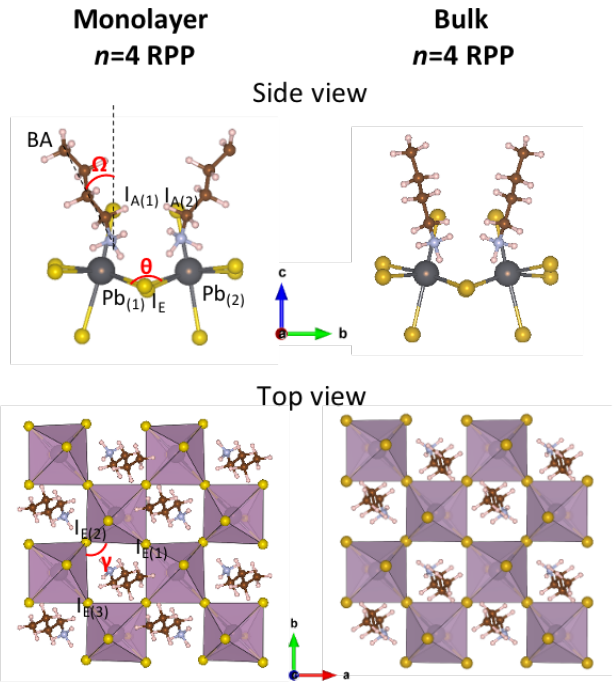

Supplementary Figure 1. The relaxed models of the top BA molecules and surface/interface octahedrons of monolayer (exfoliated) and bulk *n* = 4 RPPs.

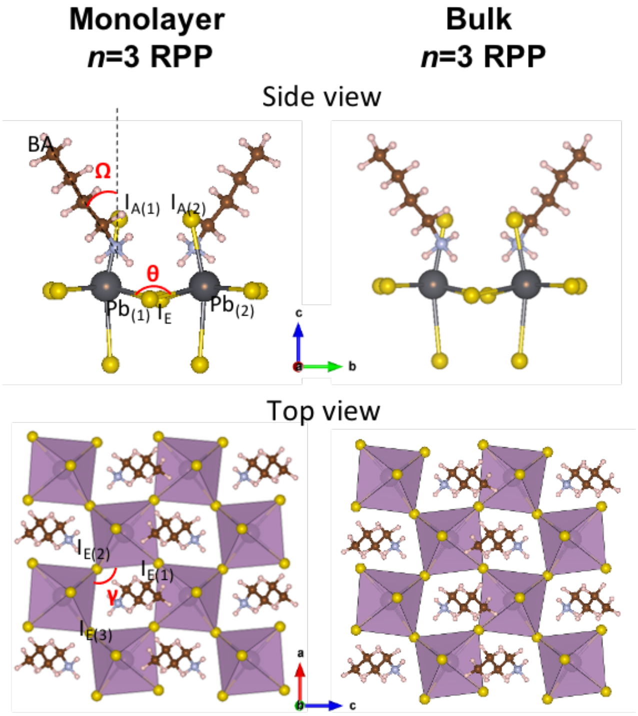

Supplementary Figure 2. The relaxed models of the top BA molecules and surface/interface octahedrons of monolayer (exfoliated) and bulk *n* = 3 RPPs.

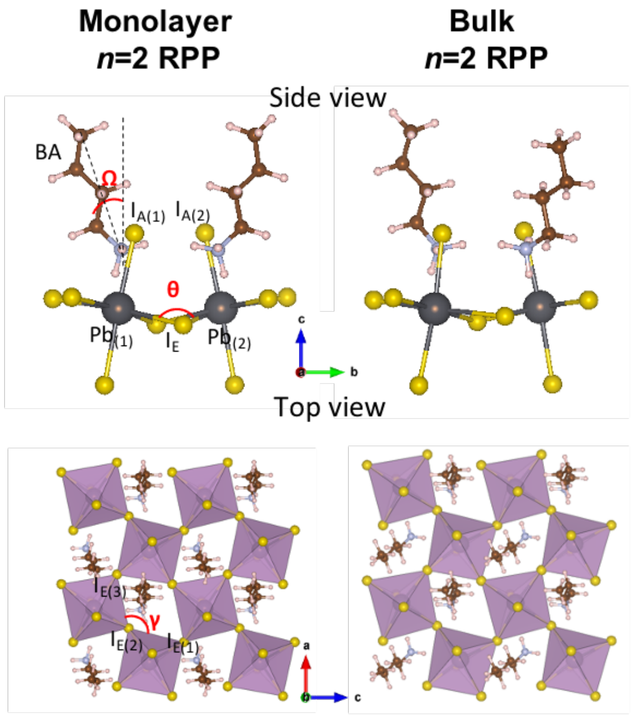

Supplementary Figure 3. The relaxed models of the top BA molecules and surface/interface octahedrons of monolayer (exfoliated) and bulk *n* = 2 RPPs.

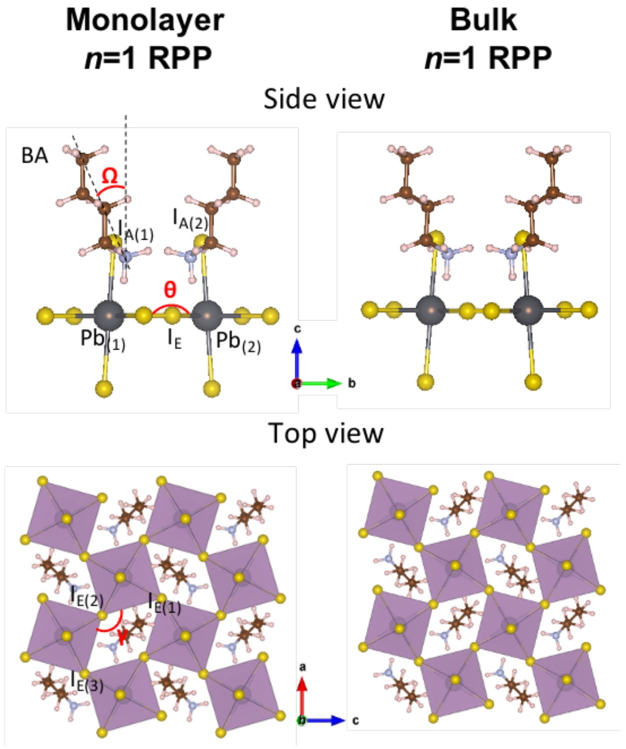

Supplementary Figure 4. The relaxed models of the top BA molecules and surface/interface octahedrons of monolayer (exfoliated) and bulk *n* = 1 RPPs.

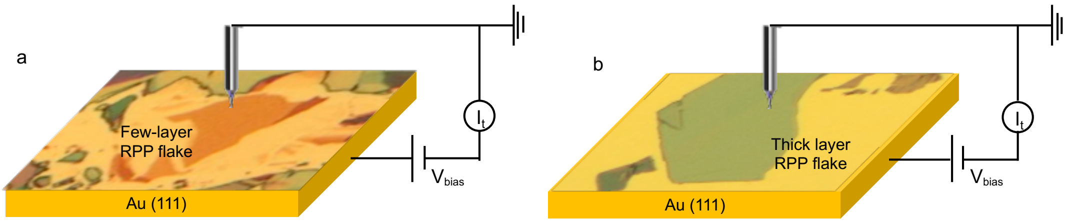

Supplementary Figure 5. Schematic illustration of the sample preparation for the STM measurement. a, Few-layer RPP flake for STM. **b**, Thick layer RPP flake for STM.

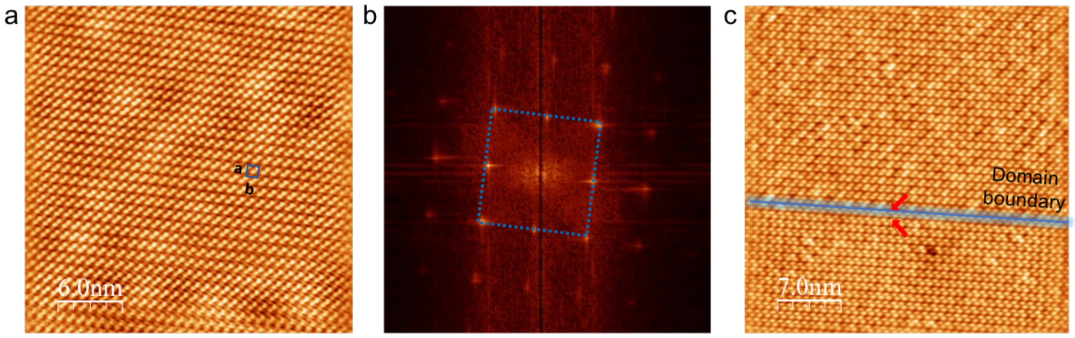

Supplementary Figure 6. Large-scale STM images of exfoliated *n* = 4 RPP. a, Typical STM image of *n* = 4 RPP in large-scale, showing the “dimer” like structure which represents the apical I atoms. **b**, Corresponding Fast Fourier Transition (FFT) image of **a**, clearly verifying the orthogonal symmetry. **c**, STM image of *n* = 4 RPP showing two domains. STM setpoint: V_{bias} = +2.3 V, I_{t} = 30 pA.

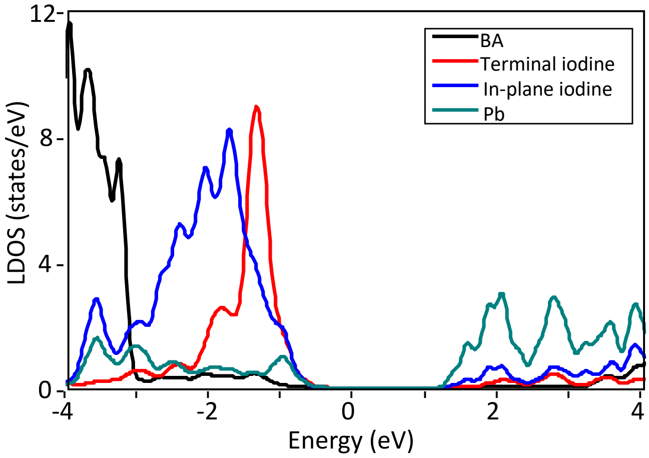

Supplementary Figure 7. Local density of states (LDOS) of monolayer *n* = 4 RPP. The empty states are mostly contributed by Pb and I atoms. BA molecules (black curve) have negligible contribution to the electron density of empty states until above +3.2 eV while they can contribute to filled states below -0.8 eV.

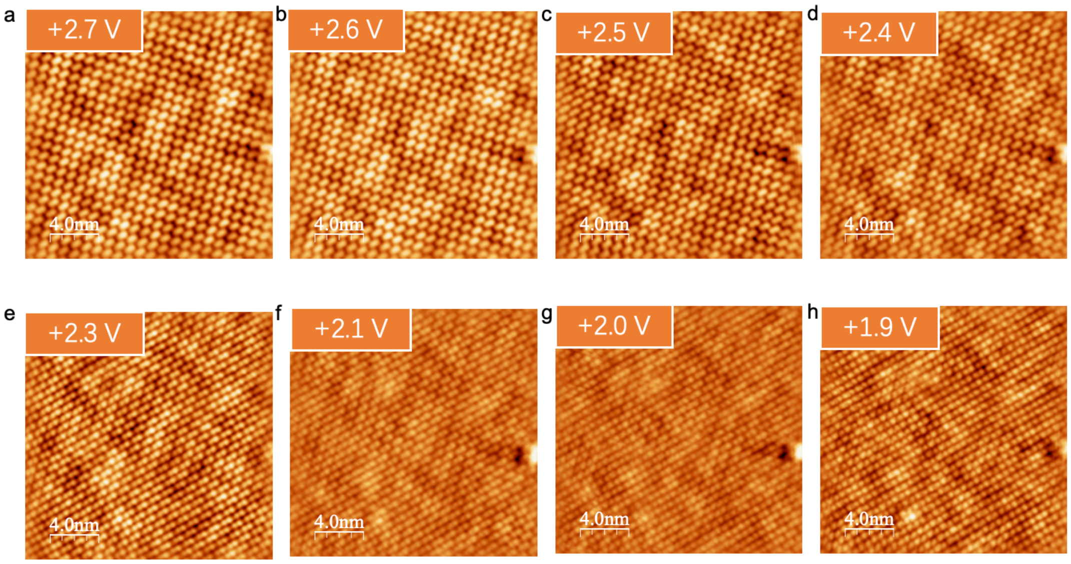

Supplementary Figure 8. STM images of *n* = 4 RPP flake at various positive bias voltages.

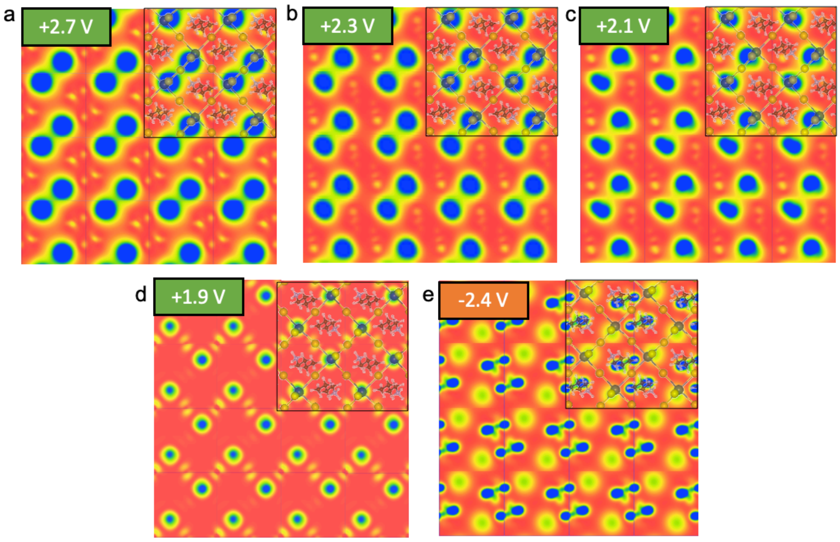

Supplementary Figure 9. STM simulations at different bias voltages of the same region on *n* = 4 RPP, showing the various electron states contribution from different atoms. (**a**) +2.7 V, (**b**) +2.3 V, (**c**) +2.1 V, apical I atoms dominate; (**d**) +1.9 V, lead atoms dominate; (**e**) -2.4 V, both BA molecules and apical I atom dominate. The correspond relaxed model are overlapped. All the STM simulations resemble the experimental STM images.

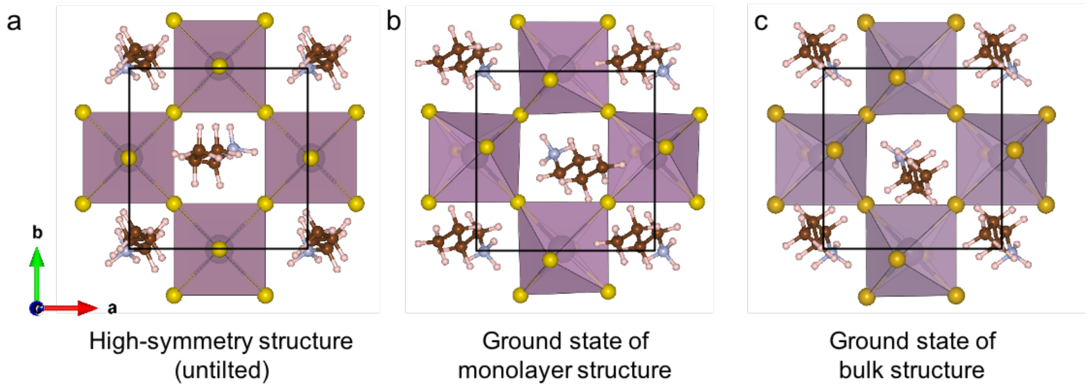

Supplementary Figure 10. Simulation models of three different structures of *n* = 4 RPP. **a**, High-symmetry (HS) structure; **b**, Ground state (GS) of monolayer (exfoliated) structure and **c**, Ground state of bulk structure. The HS structure is fixed to be totally un-tilted while the GS structures are fully relaxed.

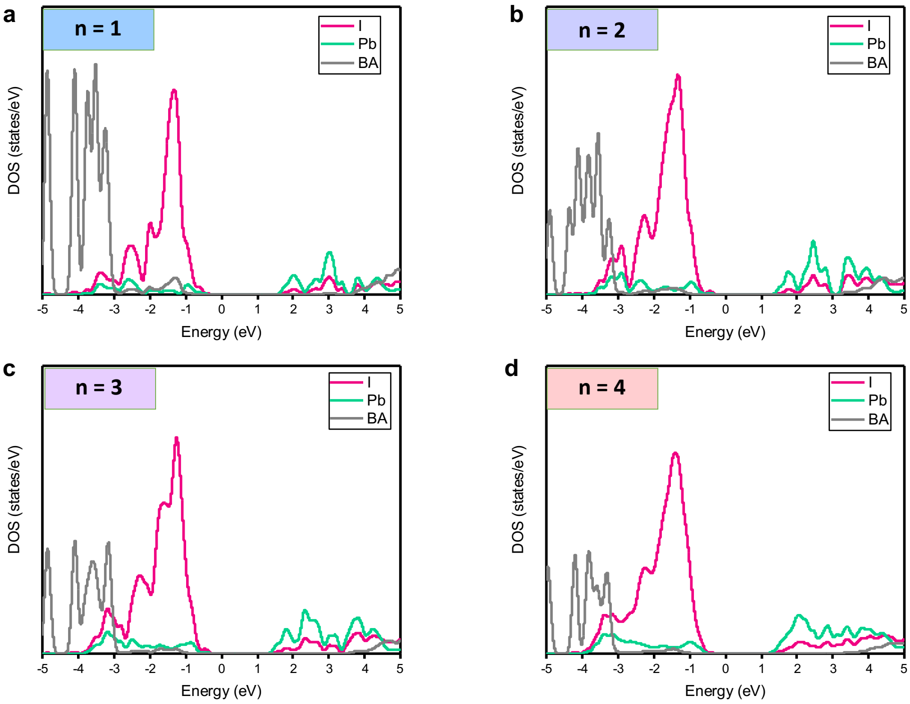

Supplementary Figure 11. Local density of states (LDOS) of *n* = 1 to *n* = 4 2D RPP $(BA)_2(MA)_{n-1}Pb_nI_{3n+1}$.

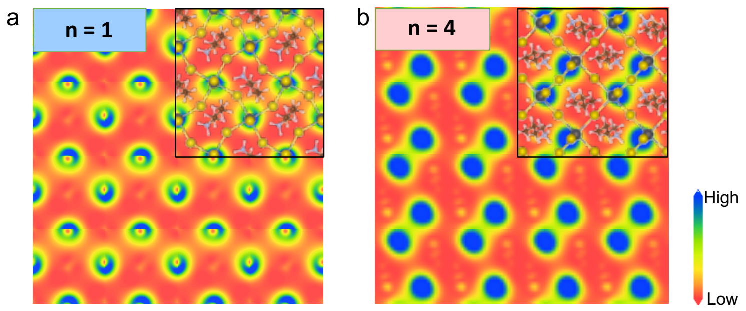

Supplementary Figure 12. STM simulations at the bias voltages of +2.3 V for *n* = 1 and *n* = 4 2D RPP $(BA)_2(MA)_{n-1}Pb_nI_{3n+1}$. The correspond relaxed model are overlapped, indicating the charge states are dominated by apical I atoms for both structures.

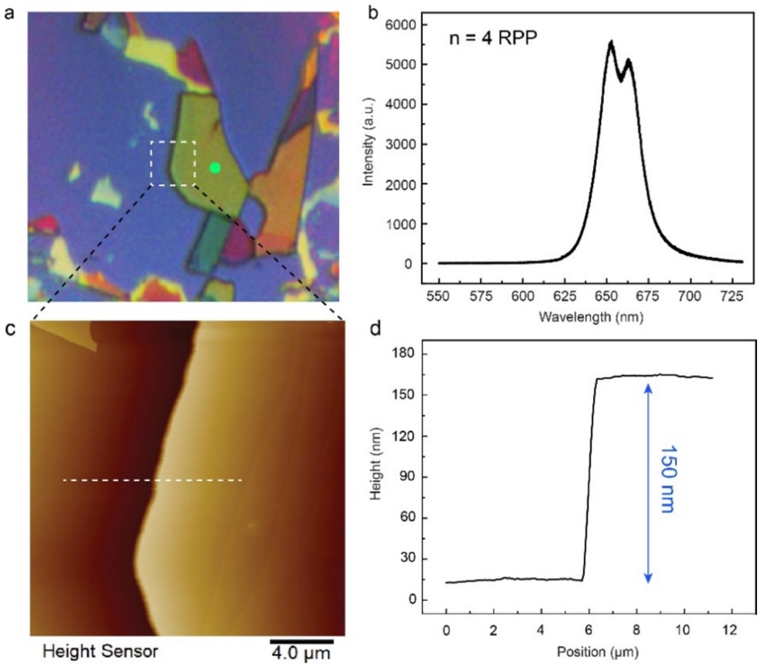

Supplementary Figure 13. a, Optical image of exfoliated *n* = 4 RPP flake for AFM scanning. **b**, Corresponding PL emission from the position of green dot indicated in **a**. **c**, AFM image of the square region in **a**. **d**, Corresponding height profiles along the dotted line in **c**.

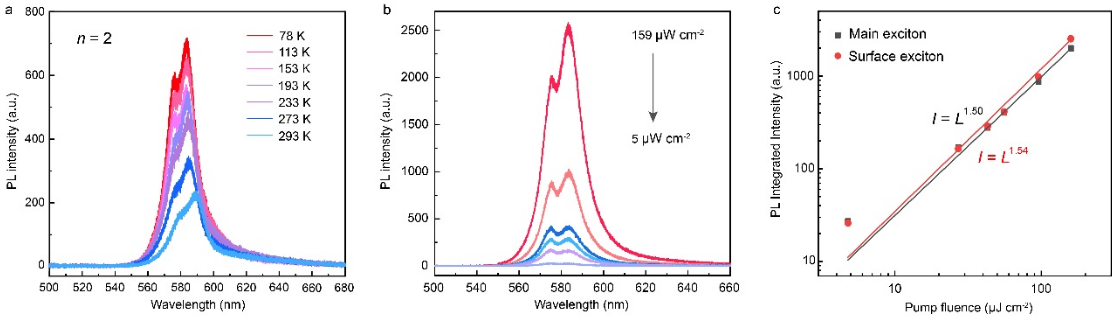

Supplementary Figure 14. The behavior of the new redshifted emission in *n* = 2 RPP. a, Temperature-dependent PL measurement in *n* = 2 RPP. **b**, Power-dependent PL measurement in *n* = 2 RPP. **c**, PL integrated intensity as function of laser power density of the two excitonic emissions in *n* = 2 RPP.

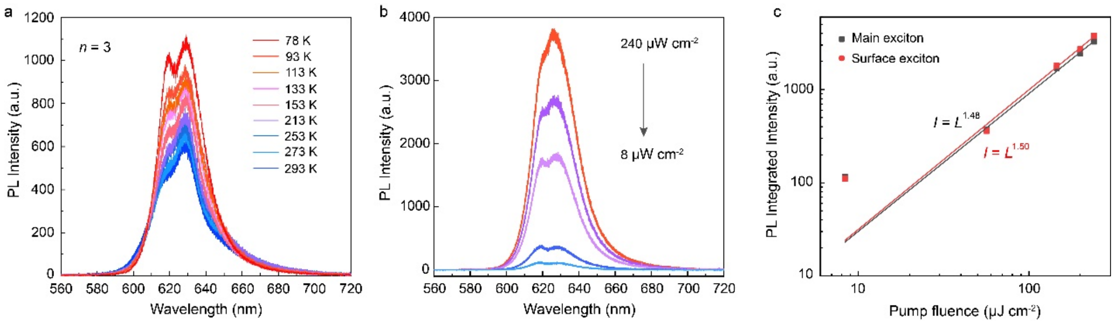

Supplementary Figure 15. The behavior of the new redshifted emission in *n* = 3 RPP. a, Temperature-dependent PL measurement in *n* = 3 RPP. **b**, Power-dependent PL measurement in *n* = 3 RPP. **c**, PL integrated intensity as function of laser power density of the two excitonic emissions in *n* = 3 RPP.

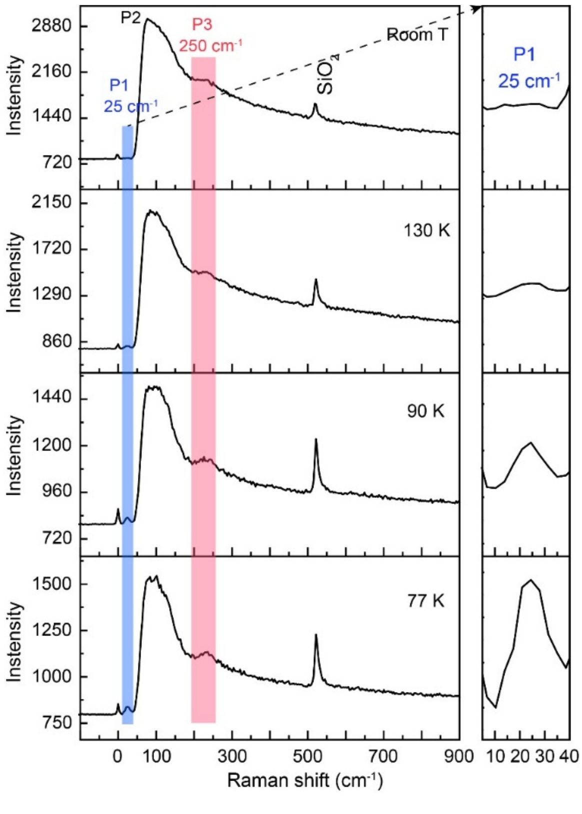

Supplementary Figure 16. Temperature-dependent Raman spectra of exfoliated *n* = 2 $(BA)_2(MA)Pb_2I_7$.

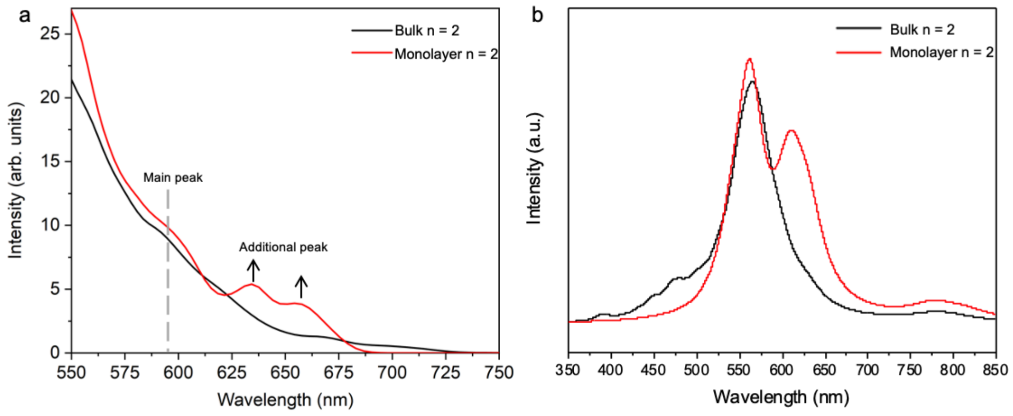

Supplementary Figure 17. DFT simulated absorption spectra of both monolayer and bulk n = 2 RPP structure by **a**, Nonadiabatic molecular dynamic simulated method and **b**, Time-dependent DFT method. Both of the simulated optical absorption spectra show an additional emission in monolayer n = 2 RPP compared with its bulk.

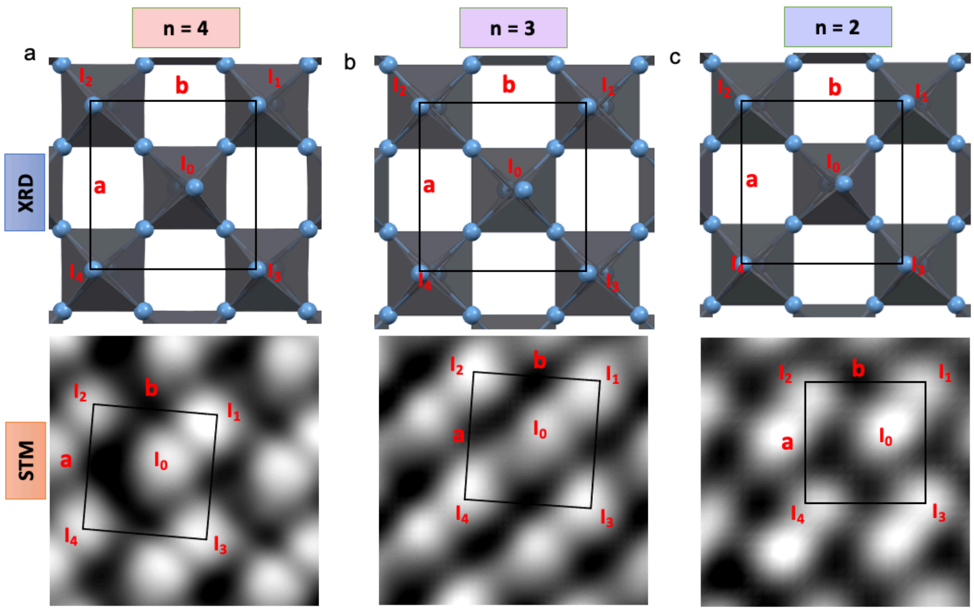

Supplementary Figure 18. Top-view illustration of $n > 1$ RPP structures from XRD (upper row) and STM (lower row) study, respectively. The unit cell is marked by the rectangle with lattice parameters a and b. I atoms are labeled for the comparison in Supplementary Table 8-10.

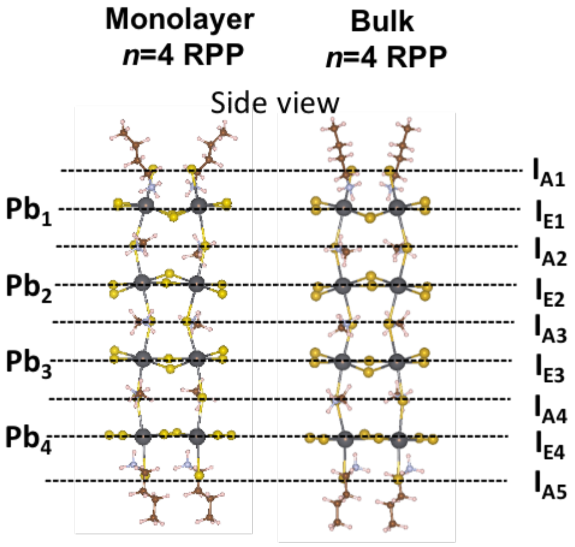

Supplementary Figure 19. The relaxed models of the whole unit cell of monolayer(exfoliated) and bulk *n* = 4 RPP structures.

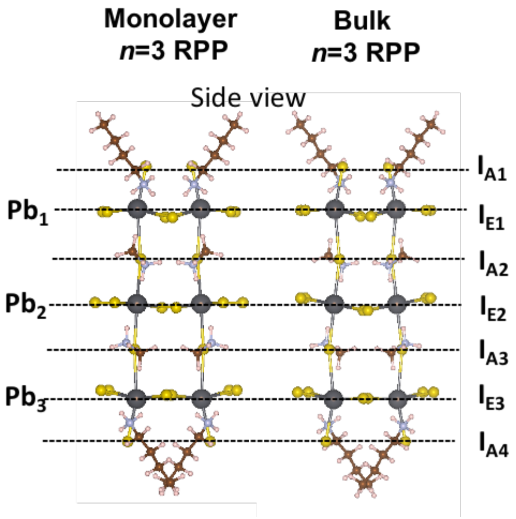

Supplementary Figure 20. The relaxed models of the whole unit cell of monolayer(exfoliated) and bulk *n* = 3 RPP structures.

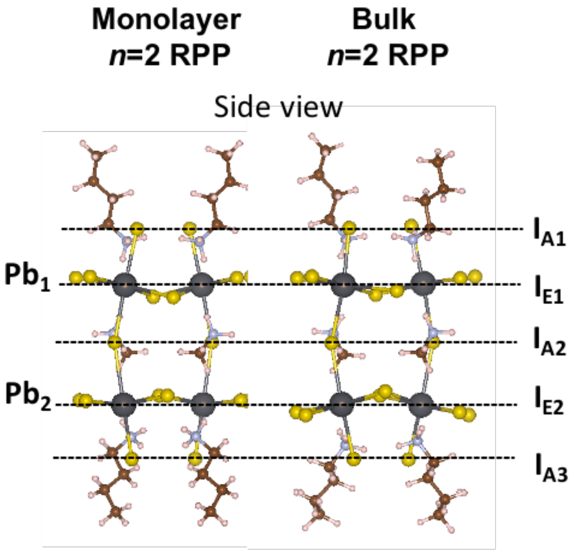

Supplementary Figure 21. The relaxed models of the whole unit cell of monolayer(exfoliated) and bulk n **= 2 RPP structures.**

Supplementary Table 1. Summary of former studies relating to structural distortions in 2D hybrid perovskites.

Reference No.	Journal	Authors	Key findings	Techniques used to study structural distortion
1	ACS Nano	Matan et. al	Probe the evolution of the structural dynamics with temperature across the phase transition of $(BA)_2PbI_4$.	Polarization-orientation (PO) Raman scattering; DFT calculations;
2	Chem. Mater.	Stoumpos, et. al	Synthesis, crystal structure, and optical characterization of the 2D $(BA)_2(MA)_{n-1}PbnI_{3n+1}$ (n = 1,2,3,4)	Single-crystal X-ray diffraction; DFT calculations;
3	Adv. Electron. Mater.	Liang et. al	Reveal branched chain molecules aid to enhance the crystallization of 2D perovskites in comparison to linear chain counterparts	Single-crystal X-ray diffraction
4	Chem. Mater.	Legein et. al	Investigate the Hydrogen and Halogen Bonding interactions at the organic-inorganic interface which affect the distortion	Single-crystal X-ray diffraction; DFT calculations;
5	Dalton Trans.	Billing et. al	Crystal structure and phase transitions of $[(C_nH_{2n+1}NH_3)_2PbI_4]$, n = 7-10.	Single-crystal X-ray diffraction
6	Inorg. Chem.	Martin et. al	Tune the bandgap of 2d $(RNH_3)_2SnI_4$ by changing the Sn-I-Sn bond angle.	DFT calculations only
7	JACS	Kanatzidis et. al	Reveal the structural distortions can lead to a white-light emission in 2d $EA_4Pb_3Cl_{10}$	Powder and Single Crystal X-ray Diffraction.
8	JACS	Kanatzidis et. al	Synthesis, crystal structure, and optical and electronic properties of new type 2d DJ perovskites	Single-crystal X-ray diffraction
9	Angewandte Chemie	Luo et. al	Phase transition in 2d perovskite $(C_4H_9NH_3)_2(MA)_2Pb_3Br_{10}$ and reveal the ferroelectric effects.	Single-crystal X-ray diffraction
10	Chem. Mater.	Tisdale et.al	Synthesis, observed structural distortion and PL peak shifts across phase transitions in 2d perovskites.	Powder and Single Crystal X-ray Diffraction.
11	Chem. Mater.	Mohammed et. al	Layer-dependent Rashba effect in 2D perovskites which is associated with structural distortions.	DFT calculations only

12	Inorg. Chem.	Mitzi et. al	Reveal Pb−I−Pb bond angle between adjacent PbI_6 octahedra has a more global effect than other bond angles and lengths on the exciton properties	Single-crystal X-ray diffraction
13	JACS	Petrozza et. al	Reveal the structural distortions of the inorganic lattice play a key role in carrier localization and the subsequent broadband emission.	Single-crystal X-ray diffraction; Raman; DFT calculations;
14	Nat. Commun.	Mitzi et. al	The bond angle disparity connected with asymmetric tilting distortions breaks local inversion symmetry and strongly correlates with spin-splitting in 2d perovskites.	DFT calculations only

Supplementary Table 2. Summary of former studies relating to structural distortions in 3D hybrid perovskites

Reference No.	Journal	Authors	Key findings	Techniques used to study structural distortion
1	Nat. Commun.	Giustino et.al	Octahedral tilting angles are strongly correlating with the optical band gap in 3d perovskites	DFT calculations only
2	Adv. Mater.	Xiao et. al	Observation of order-disorder transformation of MA cations which induced phase transitions and anomalous photoluminescence.	Single Crystal Neutron Diffraction Temperature-Dependent X-ray Diffraction
3	Chem. Comm.	Cheetham et. al	Reveal the strong hydrogen-bonding interaction with MA molecules and the tilting of the PbI_6 octahedra.	DFT calculations only
4	Chem. Comm.	Kieslich et. al	Investigate the variable temperature (100–450 K) and high pressure phase transition in $[(NH_2)_2CH]PbI$	Single crystal X-ray diffraction; molecular dynamics (MD) simulations; DFT calculations
5	Chem. Mater.	Jang et. al	Reveal the hydrogen bonding between an organic A-cation and the halide frame plays a significant role in octahedral tilting of $MAPbI_3$	DFT calculations only

			perovskite.	
6	JACS	McGehee et. al	Identify two competing mechanisms through which A-site cation influences the band gap of 3D hybrid perovskites.	Single-crystal X-ray diffraction;<br>DFT calculations;
7	Nano Lett.	Angelis et. al	Organic A-cation can tune the structure distortion and spin-obit coupling by the interplay of size effects and hydrogen bonding.	DFT calculations only
8	Nat. Mater.	Delaire et. al	Reveal structural instabilities and large atomic fluctuations that impact optical and thermal properties in $CsPbBr_3$	Single Crystal Neutron Diffraction;<br>Single-crystal X-ray diffraction;<br>DFT calculations;
9	Phy. Rev. Lett.	Ross et. al	Reveal a general rule for predicting the variation in transition temperatures of tilt transitions in perovskite.	DFT calculations only

Supplementary Table 3. Bond angles and bond lengths of monolayer (exfoliated) and bulk *n* = 4 RPPs, as indicated in Supplementary Fig. 1.

		Monolayer (exfoliated) *n* = 4 RPP	Bulk *n* = 4 RPP
**Bond Angle (^{o})**	BA tilt (Ω)	27.1	14.4
	$Pb_{(1)}$-I_E-$Pb_{(2)}$: θ	145.3	150.5
	Out-of-plane tilt: Δθ	34.7	29.5
	$I_{E(1)}$-$I_{E(2)}$-$I_{E(3)}$: γ	93.2	88.4
	In-plane tilt: Δγ	3.2	1.6
**Bond Length (Å)**	$I_{A(1)}$-$I_{A(2)}$	4.58	4.75
	$Pb_{(1)}$-$Pb_{(2)}$	6.09	6.09
	$Pb_{(1/2)}$-$I_{A(1/2)}$	3.09/3.05	3.09/3.07
	$Pb_{(1/2)}$-I_E	3.20/3.18	3.16/3.16

Supplementary Table 4. Bond angles and bond lengths of monolayer(exfoliated) and bulk *n* = 3 RPPs, as indicated in Supplementary Fig. 2.

		Monolayer (exfoliated) *n* = 3 RPP	Bulk *n* = 3 RPP
**Bond Angle (^{o})**	BA tilt (Ω)	33.9	34.9
	$Pb_{(1)}$-I_E-$Pb_{(2)}$: θ	158.1	161.2
	Out-of-plane tilt: Δθ	21.9	18.8
	$I_{E(1)}$-$I_{E(2)}$-$I_{E(3)}$: γ	102.3	106.7
	In-plane tilt: Δγ	12.3	16.7
**Bond Length (Å)**	$I_{A(1)}$-$I_{A(2)}$	5.47	5.52
	$Pb_{(1)}$-$Pb_{(2)}$	6.25	6.22
	$Pb_{(1/2)}$-$I_{A(1/2)}$	3.04; 3.04	3.05; 3.05
	$Pb_{(1/2)}$-I_E	3.23; 3.18	3.23; 3.17

Supplementary Table 5. Bond angles and bond lengths of monolayer(exfoliated) and bulk *n* = 2 RPPs, as indicated in Supplementary Fig. 3.

		**Monolayer (exfoliated) *n* = 2 RPP**	**Bulk *n* = 2 RPP**
**Bond Angle (º)**	BA tilt (Ω)	19.5; 17.5	13.3; 16.1
	$Pb_{(1)}$-I_E-$Pb_{(2)}$: θ	155.3	168.5
	Out-of-plane tilt: Δθ	24.7	11.5
	$I_{E(1)}$-$I_{E(2)}$-$I_{E(3)}$: γ	115.5	112.8
	In-plane tilt: Δγ	25.5	22.8
**Bond Length (Å)**	$I_{A(1)}$-$I_{A(2)}$	5.29	5.36
	$Pb_{(1)}$-$Pb_{(2)}$	6.14	6.34
	$Pb_{(1/2)}$-$I_{A(1/2)}$	3.19; 3.20	3.14; 3.22
	$Pb_{(1/2)}$-I_E	3.24; 3.25	3.23; 3.33

Supplementary Table 6. Bond angles and bond lengths of monolayer(exfoliated) and bulk *n* = 1 RPPs, as indicated in Supplementary Fig. 4.

		**Monolayer (exfoliated) *n* = 1 RPP**	**Bulk *n* = 1 RPP**
**Bond Angle (º)**	BA tilt (Ω)	22.6	21.6
	$Pb_{(1)}$-I_E-$Pb_{(2)}$: θ	178.9	178.5
	Out-of-plane tilt: Δθ	1.1	1.5
	$I_{E(1)}$-$I_{E(2)}$-$I_{E(3)}$: γ	122.2	118.2
	In-plane tilt: Δγ	32.2	28.2
**Bond Length (Å)**	$I_{A(1)}$-$I_{A(2)}$	5.84	5.88
	$Pb_{(1)}$-$Pb_{(2)}$	6.13	6.21
	$Pb_{(1/2)}$-$I_{A(1/2)}$	3.24	3.24
	$Pb_{(1/2)}$-I_E	3.21	3.21

Supplementary Table 7. Bond angles and bond lengths of bulk; monolayer; h-BN covered monolayer *n* = 2 RPPs, as indicated in Fig. 5d-5f.

		Bulk<br>*n* = 2 RPP	Monolayer<br>*n* = 2 RPP	h-BN covered monolayer *n* = 2 RPP
Bond Angle (°)	BA tilt (Ω_1; Ω_2)	13.3; 16.1	19.5; 17.5	26.9; 26.6
	Out-of-plane tilt ($\Delta\theta$):	11.5	24.7	12.4
	In-plane tilt($\Delta\gamma$):	22.8	25.5	27.5
Bond Length (Å)	$I_{A(1)}$-$I_{A(2)}$	5.36	5.29	5.73
	$Pb_{(1)}$-$Pb_{(2)}$	6.34	6.14	6.26
	$Pb_{(1/2)}$-$I_{A(1/2)}$	3.14; 3.22	3.19; 3.20	3.20; 3.19
	$Pb_{(1/2)}$-I_E	3.23; 3.33	3.24; 3.25	3.25; 3.27

Supplementary Table 8. Comparison of surface structure of exfoliated *n* = 4 RPP by STM with bulk structure by XRD studies, as indicated in Supplementary Fig. 18a.

n = 4 RPP structure		STM on exfoliated RPP	XRD on bulk RPP
Atom distance (Å)	a	8.90	8.93
	b	8.51	8.88
	I_0-I_1	4.58	5.61
	I_0-I_2	6.87	7.07
	I_0-I_3	5.71	5.61
	I_0-I_4	7.12	7.07

Supplementary Table 9. Comparison of surface structure of exfoliated *n* = 3 RPP by STM with bulk structure by XRD studies, as indicated in Supplementary Fig. 18b.

n = 3 RPP structure		STM on exfoliated RPP	XRD on bulk RPP
Atom distance (Å)	a	9.13	8.93
	b	8.71	8.88
	I_0-I_1	5.02	5.59
	I_0-I_2	6.74	7.09

	I_0-I_3	6.34	5.59
	I_0-I_4	6.89	7.09

Supplementary Table 10. Comparison of surface structure of exfoliated *n* = 2RPP by STM with bulk structure by XRD studies, as indicated in Supplementary Fig. 18c.

***n* = 2 RPP structure**		**STM on exfoliated RPP**	**XRD on bulk RPP**
**Atom distance (Å)**	a	8.80	8.95
	b	8.73	8.86
	I_0-I_1	4.85	5.59
	I_0-I_2	6.46	7.07
	I_0-I_3	5.42	5.59
	I_0-I_4	7.05	7.07

Supplementary Table 11. Bond angles and bond lengths of the whole unit cell of monolayer (exfoliated) and bulk *n* = 4 RPP structures, as indicated in Supplementary Fig. 19.

		**Monolayer (exfoliated) *n* = 4 RPP**	**Bulk *n* = 4 RPP**
**Bond Angle (°)**	Pb_1-I_{E1}-Pb_1	145.3	150.5
	Pb_2-I_{E2}-Pb_2	179.0	179.2
	Pb_3-I_{E3}-Pb_3	179.5	177.0
	Pb_4-I_{E4}-Pb_4	176.1	174.2
	Pb_1-I_{A2}-Pb_2	150.2; 157.8	154.7; 150.7
	Pb_2-I_{A3}-Pb_3	153.3; 146.7	149.7; 151.2
	Pb_3-I_{A4}-Pb_4	161.6; 164.5	156.6; 165.5
**Bond Length (Å)**	Pb_1-I_{A1}	3.09; 3.05	3.09; 3.07
	Pb_1-I_{A2}	3.30; 3.36	3.32; 3.35
	Pb_2-I_{A2}	3.19; 3.16	3.17; 3.18
	Pb_2-I_{A3}	3.17; 3.19	3.17; 3.18
	Pb_3-I_{A3}	3.18; 3.18	3.20; 3.20

Pb_3-I_{A4}	3.17; 3.17	3.15; 3.16
Pb_4-I_{A4}	3.23; 3.25	3.27; 3.23
Pb_4-I_{A5}	3.18; 3.17	3.15; 3.17
Pb_1-I_{E1}	3.20; 3.18	3.16; 3.16
Pb_2-I_{E2}	3.19; 3.16	3.17; 3.16
Pb_3-I_{E3}	3.16; 3.16	3.14; 3.15
Pb_4-I_{E4}	3.20; 3.23	3.17; 3.28

Supplementary Table 12. Bond angles and bond lengths of the whole unit cell of monolayer(exfoliated) and bulk *n* = 3 RPP structures, as indicated in Supplementary Fig. 20.

		**Monolayer (exfoliated) *n* = 3 RPP**	**Bulk *n* = 3 RPP**
**Bond Angle (°)**	Pb_1-I_{E1}-Pb_1	158.1	161.2
	Pb_2-I_{E2}-Pb_2	177.5	164.3
	Pb_3-I_{E3}-Pb_3	173.8	177.3
	Pb_1-I_{A2}-Pb_2	173.5; 173.6	168.4; 168.4
	Pb_2-I_{A3}-Pb_3	165.7; 165.9	168.4; 168.4
**Bond Length (Å)**	Pb_1-I_{A1}	3.04; 3.04	3.05; 3.05
	Pb_1-I_{A2}	3.46; 3.46	3.57; 3.57
	Pb_2-I_{A2}	3.17; 3.17	3.22; 3.22
	Pb_2-I_{A3}	3.20; 3.20	3.22; 3.22
	Pb_3-I_{A3}	3.48; 3.48	3.57; 3.57
	Pb_3-I_{A4}	3.05; 3.05	3.05; 3.05
	Pb_1-I_{E1}	3.23; 3.18	3.23; 3.17
	Pb_2-I_{E2}	3.30; 3.30	3.22; 3.16
	Pb_3-I_{E3}	3.22; 3.15	3.22; 3.17

Supplementary Table 13. Bond angles and bond lengths of the whole unit cell of monolayer(exfoliated) and bulk *n* = 2 RPP structures, as indicated in Supplementary Fig. 21.

		Monolayer (exfoliated) $n = 2$ RPP	Bulk $n = 2$ RPP
Bond Angle (°)	Pb_1-I_{E1}-Pb_1	155.3	168.5
	Pb_2-I_{E2}-Pb_2	160.5	153.2
	Pb_1-I_{A2}-Pb_2	161.1; 161.5	159.7; 158.0
Bond Length (Å)	Pb_1-I_{A1}	3.19; 3.20	3.14; 3.22
	Pb_1-I_{A2}	3.23; 3.24	3.26; 3.26
	Pb_2-I_{A2}	3.51; 3.51	3.51; 3.40
	Pb_2-I_{A3}	3.06; 3.06	3.08; 3.08
	Pb_1-I_{E1}	3.24; 3.25	3.23; 3.33
	Pb_2-I_{E2}	3.16; 3.21	3.15; 3.22

Supplementary Discussion

Discussion on DFT calculations of surface relaxation on bulk and monolayer (exfoliated) *n* = 1 to *n* = 4 RPPs.

With the splicing of the interdigital organic bilayers during mechanical exfoliation to generate fresh surface on RPP crystals, the remaining one-layer organic cation in top begins to relax, induces subsequent structural adjustment that extends to the sub-surface. To gain insight into this, Ab-initio calculations were performed to investigate how the geometrical structures changes from the bulk to exfoliated layers, and systematic studies were carried out from n = 1 to n = 4 RPPs. The bulk system was constructed with two unit cells based on the lattice parameters from XRD results in the former study. The exfoliated surface was simulated as monolayer structure since they both have relaxed organic layer on top, which was generated by slicing the middle of the interdigital organic layers to one unit cell consisting of two organic molecular layers sandwiching the inorganic layer in the middle with a vacuum thickness of 15Å to avoid interactions.

RPP n = 4 $(C_4H_9NH_3)_2MA_3Pb_4I_{13}$

We first analysed the change of bond angles and bond lengths of the surface octahedrons as well as the configuration of top BA organic between bulk and monolayer (exfoliated) n = 4 RPP structures. The relaxed models are illustrated in Supplementary Fig. 1, the tilt angle involving BA chain is marked as Ω, which changes from 14.4° to 27.1° between bulk and monolayer. The surface octahedral tilt is analysed on the basis of out-of-plane ($\Delta\theta$) and in-plane tilt ($\Delta\gamma$), which are investigated by the changes of particular bond angles (θ and γ) with respect to the untilted structure. According to our models, both the out-of-plane tilt and in-plane tilt angles are enhanced by 5.2° and 1.6° respectively from bulk to monolayer. Detailed bond angles of bulk and monolayer structure are shown in Supplementary Table 3. Compared with the bond angles, the change of bond lengths is negligible. The equatorial bond length ($Pb\text{-}I_E$) in the monolayer is increased by 0.02~0.04 Å whereas the axial bond length ($Pb\text{-}I_A$) is almost unchanged. The apical I-I distance is decreased from 4.75 Å to 4.58 Å in monolayer due to the apparently enhanced out-of-plane tilt.

RPP n = 3 $(C_4H_9NH_3)_2MA_2Pb_3I_{10}$

Similar to n = 4 RPP, we analysed the change of bond angles and bond lengths of the surface octahedrons as well as the configuration of top BA organic between bulk and monolayer (exfoliated) n = 3 RPP structures. The relaxed models are illustrated in Supplementary Fig. 2. The out-of-plane tilt and in-plane tilt angles are enhanced by 3.1° and weaken by 4.4° respectively from bulk to monolayer. Detailed bond angles of bulk and monolayer structure are shown in Supplementary Table 4. The bond lengths are almost unchanged. The apical I-I distance is decreased slightly from 5.52 Å to 5.47 Å in monolayer due to the coherent movement of both out-of-plane and in-plane octahedral tilt.

RPP n = 2 $(C_4H_9NH_3)_2MA_1Pb_2I_7$

The relaxed models of bulk and monolayer (exfoliated) n = 2 RPP structures are illustrated in Supplementary Fig. 3, showing apparent structural changes. The two BA molecules in the unit

cell are not symmetric, which changes from 13.3 °/16.1° to 19.5 °/17.5° between bulk and monolayer. The out-of-plane tilt and in-plane tilt angles are enhanced by 13.2° and 2.7° respectively from bulk to monolayer. Detailed bond angles and bond lengths are shown in Supplementary Table 5. The Pb-$I_{E/A}$ bond length of two octahedrons (e.g., Pb_1-I_{E1} and Pb_2-I_{E2}) in the unit cell of bulk structure shows small deviation of 0.08~0.10 Å. However, it becomes almost the same in monolayer structure. The distance between two Pb atoms of two octahedrons decreases from 6.34 Å in the bulk to 6.14 Å in monolayer structure. The apical I-I distance is also decreased slightly from 5.36 Å to 5.29 Å in the monolayer.

RPP $n = 1$ $(C_4H_9NH_3)_2PbI_4$

The relaxed models of bulk and monolayer (exfoliated) $n = 1$ RPP structures are illustrated in Supplementary Fig. 4, showing almost unchanged structures. The BA tilt angles change by 1.0 ° and the out-of-plane tilt change by only 0.4°, however, the in-plane tilt change by 4.0 ° in the monolayer structure. Detailed bond angles and bond lengths are shown in Supplementary Table 6. Both the equatorial bond length (Pb-I_E) and the axial bond length (Pb-I_A) are almost unchanged. The distance between two Pb atom decreases from 6.21 Å to 6.13 Å and the apical I-I distance also decreases slightly from 5.88 Å to 5.84 Å in monolayer, which should be due to the change of in-plane tilt.

Sample preparation process of the 2D perovskites for the STM measurement

Single crystalline bulk 2D perovskite crystals with centimeter-size can be exfoliated into 2D thin films by mechanical-exfoliation method. The exfoliated flakes are then transferred onto Au or HOPG substrate for STM measurement. The exfoliation and transfer process were performed in an Ar-filled glove box. The sample is loaded in a vacuum transfer box into UHV chambers for STM. A long-focus camera is used to help locate the tip position. As illustrated in Supplementary Fig. 5, RPP flakes with various thickness can be distinguished and measured by STM.

Comparison of our study with former studies on the octahedral tilt in hybrid perovskites

The bulk structure of our RPP crystal is no different from the bulk structure of RPP crystals reported, and X-ray data we have already reported that in detail in the supporting information of our previous work[1]. The difference here is we are paying attention to surface structure of these crystals using STM which is the highlight of this work.

We compare the structure information of 2D RPPs obtained in our STM study with that from the reported single crystal X-ray diffraction study[2]. The detailed comparisons of lattice distances of $n > 1$ RPPs are shown in Supplementary Table 8-10, the compared atom distances measured by STM and bulk XRD are marked in supplementary Fig. 18. Compared with structures from XRD data, the STM determined structure shows the shorter apical I-I distance (I_0-I_1) at the surface, indicating obvious octahedra tilt with higher degree of out-of-plane tilt. Lower crystal symmetry is found in our STM determined structure rather than that from XRD study.

Discussion on DFT calculations of structural distortion through the whole unit cell on bulk and monolayer (exfoliated) *n* > 1 RPPs.

In addition to the surface relaxation, we discuss the structural distortion through the whole unit cell due to the synergistic movement of PbI cages of the perovskite structures. The relaxed models of the whole unit cell of bulk and monolayer structures are shown in Supplementary Fig. 19-21 from $n = 4$ to $n = 2$ RPPs. The corresponding change of bond angles and bond lengths between bulk and monolayer (exfoliated) from $n = 4$ to $n = 2$ RPPs are summarized in Supplementary Table 11-13. In $n > 1$ RPPs which have more than one layer of PbI_6 octahedrons, structural distortion is expected to decrease with distances from the surface; Pb-I octahedrons in the middle layer ($n = 3$ or 4) usually have lesser degree of out-of-plane tilt than those at surfaces.

Supplementary References:

1 Leng, K. *et al.* Molecularly thin two-dimensional hybrid perovskites with tunable optoelectronic properties due to reversible surface relaxation. *Nat. Mater.* **17**, 908-914 (2018).

2 Stoumpos, C. C. *et al.* Ruddlesden–Popper hybrid lead iodide perovskite 2D homologous semiconductors. *Chem. Mater.* **28**, 2852-2867 (2016).
