# Supplementary material for: Unlocking surface octahedral tilt in two-dimensional Ruddlesden-Popper perovskites
Source: Nat Commun. 2022 Jan 10;13:138. doi: 10.1038/s41467-021-27747-x (PMC8748742; doi:10.1038/s41467-021-27747-x)
Supplement: Supplementary file 2 — Peer Review File [file 41467_2021_27747_MOESM2_ESM.pdf]

nature portfolio

Peer Review File

Unlocking surface octahedral tilt in two-dimensional Ruddlesden-Popper perovskites

Open Access This file is licensed under a Creative Commons Attribution 4.0 International License, which permits use, sharing, adaptation, distribution and reproduction in any medium or format, as long as you give appropriate credit to the original author(s) and the source, provide a link to the Creative Commons license, and indicate if changes were made. In the cases where the authors are anonymous, such as is the case for the reports of anonymous peer reviewers, author attribution should be to 'Anonymous Referee' followed by a clear attribution to the source work. The images or other third party material in this file are included in the article's Creative Commons license, unless indicated otherwise in a credit line to the material. If material is not included in the article's Creative Commons license and your intended use is not permitted by statutory regulation or exceeds the permitted use, you will need to obtain permission directly from the copyright holder. To view a copy of this license, visit http://creativecommons.org/licenses/by/4.0/.

REVIEWER COMMENTS

Reviewer #1 (Remarks to the Author):

This is an extremely interesting paper regarding the local structure of 2D perovskite materials. Actually, I guess this could one of the very first paper clearly illustrated the local structure distortion in 2D perovskite both experimentally and theoretically. Such a topic is essential to understand some fundermental photophysics phenomenon of such material. The utilization of STM to characterize the surface structure is attractive and really relevant. Especially the local tilting of the octahedra in 2D layers alongside the spacing cations is important to understand the excited state dynamics or polaron formation in the community. Therefore I fully endorse the novelty and significance of this paper. The data presentation and interpretation is also systemtic and thorough to me. Therefore I think this paper can attract the interest from potential readers in Nature communication. However, the following concerns from me need to be stressed as it influence the clarification of the finding:
1) STM is a perfect method to visualize the surface structure of the extrafolied 2D perovskite flakes. However as also mentioned by the authors, different biases need to be applied in order to overcome the work function of the materials to trigger the tunneling current, and it is also associated with the atomic orbital level of each element. I wonder if such bias can also be influenced by the n value as different n values would results in the different vertical resistance will can tune the surface potential even at the same bias. If so, how to calibrate the applied bias among different samples to ensure the same species of atoms to be visualized during the measurement?
2) The measured d-spacing in figure 3 is not monotonously decreased with the n-value. Can this be explained.
3) The origins of the red emission in 2D perovksites remains to be under debate in the community. However, the most popular explanation is due to the edge state where the local structures at the edge facet should be different from the bulk structrure. As the microscopic structure of the flake has already been provided in Fig. 5. I think the author should put more explaination to exclude other possible origins of the red emission, such as the edge emission or reabsorption of the emission light, etc.
4) Would the applied bias during the STM measurement change the local structure specially the surface structure? Such possibility should also be excluded as it is important for the analysis of the surface lattice.

Reviewer #2 (Remarks to the Author):

Review “Unlocking surface octahedral tilt in two-dimensional Ruddlesden-Popper perovskites”

The manuscript describes a combined experimental and computational study of the tilting of octahedra in bulk and exfoliated quasi 2D perovskites. The main novelty is the characterization of the tilting of octahedra on the surface of the layers, which is different from the bulk. Attempts are made to correlate this with the observed optical properties. While the STM characterization of the tilting at the surface is new (as far as I know) I would rate the conceptual novelty of the presented work quite low. Distortions in perovskites (both 2D and 3D) are very well documented, both computationally and experimentally, primarily by X-ray. The latter means that there is mainly bulk information available and that the surface information obtained from STM is novel, but the differences observed are hardly surprising.
- The connection that is made with the optical properties is quite weak: would DFT simulations (e.g. TDDFT) show the two different exciton peaks originating from different places in the film?
- It has been shown by confocal microscopy for exfoliated films that differences in emission are observed in the center of the flake and around the edges, is this the case for these fluorescence measurements?
- There is no information about the thickness of the flakes (number of monolayers). This can affect the optical and structural properties significantly. The same is true for the interaction with the substrate, but in the calculations the substrate is not taken into account. The hBN calculation show

already the strong effect that can be expected here.
- Was the full exfoliation process done in inert atmosphere? Were the samples ever in contact with air during processing and measurements? In particular exfoliated BA-based layers are exceptionally prone to degradation.
- The distinction between calculations and experimental results is not always clear and should be clearly indicate din the captions of figures.
- Given the abundance of X-ray data (and previous computational work) a comparison with that structural info would be in place.

Considering these shortcomings and the lack of a clear conceptual novelty I do not recommend publication in Nature Commun.

Reviewer #3 (Remarks to the Author):

The manuscript “Unlocking surface octahedral tilt in two-dimensional Ruddlesden-Popper perovskites” is very well written and structured, where they explained and justified the claims (i.e., octahedral tilt, formation of dimmers, etc) with solid proofs by authors. This is an exciting study, where they experimentally evidenced the octahedral tilts in RPPs (n = 1 to n = 4) and visualized it through STM. Also, the theoretical calculation supports the claimed result. It is interesting to see that, the different atoms can be visualized from the 2D-RPP through STM by different applied potentials. The layer dependant exciton-phonon study was also presented in a scientific manner, and the Rashba effect also supported them with DFT.

Therefore, this manuscript has recommended to accept after consideration of the fulfillment of the following major comments,

1. Why does the in-plane octahedral tilt show less co-relation as compared to out of plane surface?
2. What is the significance of covering monolayers by h-BN? Why not other 2D materials? A minor discussion is lacking about the advantages of h-BN.
3. The fitting of figure 4d, could be much better than the existing one. Hence, the exponent may show different values.
4. Exciton-phonon coupling has shown with PL, however, it can be shown more prominently with Raman spectroscopy, since the organic cation is polar. If possible, authors can perform the Raman spectra, and can involve these results in the main or supplementary manuscript.
5. Perovskite sample preparation for the STM measurement is quite tricky but crucial to obtain fine images. Could you mention the details of the sample preparations in the supplementary portion? So that majority of researchers can be benefitted and get enough idea of sample preparation of the kind of perovskite for SPM measurements.

Point-by-point Response Letter

Please find the response letter and revised manuscript for your consideration. We are grateful for the extensive and thoughtful review provided by the reviewers, which have led us to perform additional experiments and reorganize our manuscript.

To address the reviewers' concerns, we have listed their comments and our answers/actions below in blue. The modifications to the text of the original manuscript have been highlighted in red.

I hope you will find the responses and revised manuscript satisfied.

Yours sincerely,

Dr. Kai Leng
Prof. Kian Ping Loh

Response to reviewer 1

Reviewer #1 (Remarks to the Author)

This is an extremely interesting paper regarding the local structure of 2D perovskite materials. Actually, I guess this could one of the very first paper clearly illustrated the local structure distortion in 2D perovskite both experimentally and theoretically. Such a topic is essential to understand some fundamental photo-physics phenomenon of such material. The utilization of STM to characterize the surface structure is attractive and really relevant. Especially the local tilting of the octahedra in 2D layers alongside the spacing cations is important to understand the excited state dynamics or polaron formation in the community. Therefore I fully endorse the novelty and significance of this paper. The data presentation and interpretation is also systematic and thorough to me. Therefore I think this paper can attract the interest from potential readers in Nature communication. However, the following concerns from me need to be stressed as it influence the clarification of the finding:

We thank the reviewer for the recognition of the novelty and great support to the manuscript. The point-by-point response to the reviewer #1's concerns are as follows:

1. STM is a perfect method to visualize the surface structure of the exfoliated 2D perovskite flakes. However as also mentioned by the authors, different biases need to be applied in order to overcome the work function of the materials to trigger the tunneling current, and it is also associated with the atomic orbital level of each element. I wonder if such bias can also be influenced by the n value as different n values would results in the different vertical resistance will can tune the surface potential even at the same bias. If so, how to calibrate the applied bias among different samples to ensure the same species of atoms to be visualized during the measurement?

ANS: We thank the reviewer for this comment. STM probes the local density of states of the sample at specific bias voltage. Therefore, it's possible for us to probe different atomic orbitals by applying different bias voltages due to their different electron states contributions, such as what we have discussed for n = 4 RPP in the manuscript (Fig. 2d-g). The reviewer raises the concern that the electron states distribution from the atoms may be different from n = 1 to n = 4 RPPs at the same bias applied, leading to a different interpretation of STM images. In order to determine the bias-dependence and dimensionality dependence of these STM measurements, we conducted DFT calculations of the local density-of-states (LDOS) and corresponding STM simulations for n = 1 to n = 4 RPPs.

Figure R1 shows the calculated LDOS from n = 1 to n = 4 RPPs, we found the electron states from different atom orbitals are not influenced by the dimensionality (n values) despite the band gap differences. For the conduction bands, electron states are mainly contributed by Pb 6p and I 5s orbitals at the lower energy range (< 2.8 eV), and not from the BA organics. Therefore, at the positive substrate bias (+2.3 V) used for the STM measurements of n = 1 to n = 4 RPPs (Fig.3d-g in the manuscript), the images originate from electron tunneling into the conduction bands contributed by Pb and I atoms. In the corner-sharing PbI_6 octahedral cage, the Pb atoms are at a lower position with respect to the apical iodine atoms, thus the apical I atoms are mainly imaged in STM due to the exponential dependence of the tunneling current on the tip-sample distance.

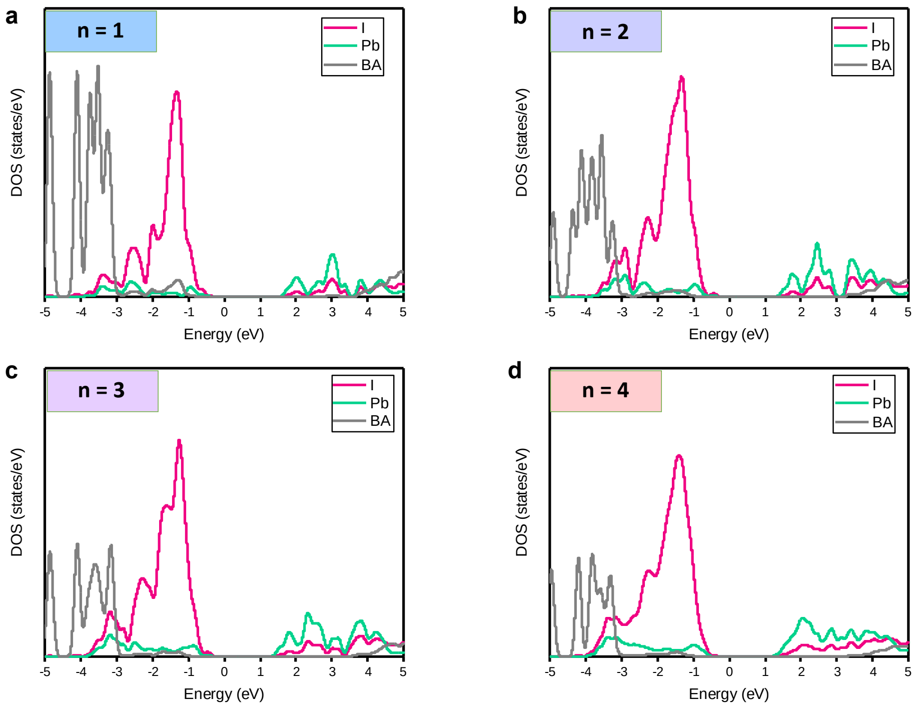

Fig. R1 | Local density of states (LDOS) of n = 1 to n = 4 2D RPPs $(BA)_2(MA)_{n-1}Pb_nI_{3n+1}$.

We also simulated the STM images at +2.3 V bias for both n = 1 and n = 4 RPPs to verify that apical I atoms are the one visualized. The simulation results are shown as Fig. R2, which indicates that the charge states from apical I atoms are dominant, agreeing well with

experimental STM images. **Therefore, we can safely conclude that our STM images at various bias (+2.3 V) are visualizing the same species of apical I atoms for n = 1 to n = 4 RPPs.**

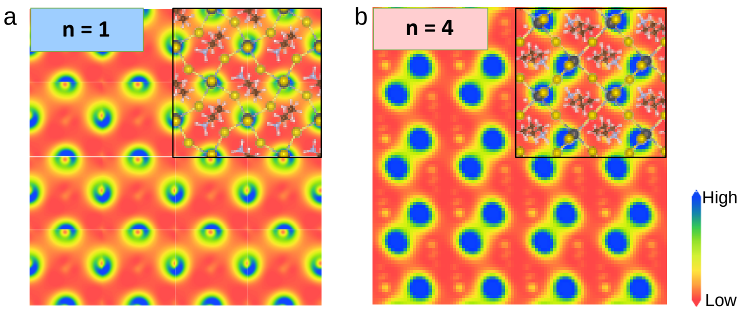

Fig. R2 | STM simulations at the bias voltages of +2.3 V for n = 1 and n = 4 2D RPPs $(BA)_2(MA)_{n-1}Pb_nI_{3n+1}$. The correspond relaxed model are overlapped, indicating the charge states are dominated by apical I atoms for both.

Action: We have added DFT calculations of the local density-of-states (LDOS) and corresponding STM simulations for n = 1 to n = 4 RPPs to the revised supplemental information (as Supplementary Figure 10 and 11). We also added a sentence to discuss the interpretation of STM images on different n values RPPs into Page 7 of the revised manuscript as follows:

"All the bright protrusions are due to apical I atoms from n = 1 to n = 4 RPPs, which has been verified by the DFT calculation of LDOS and corresponding STM image simulations (Supplementary Figure 10 and 11)."

2. The measured d-spacing in figure 3 is not monotonously decreased with the n-value. Can this be explained.

ANS: This is the most interesting aspect of the paper which reflects that we are really studying the delicate balance of internal forces near the surface of the crystal. The d-spacing refers to the distance between two nearest apical I atoms, which can be directly visualized by STM as an important descriptor for the octahedral tilting. Interestingly this distance does not decrease monotonically with increasing n values, such as the distance in n = 2 is slightly shorter than that in n = 3 RPP, which is verified by both STM and calculation models. In the text, we explained this trend as arising from balancing the Coulombic forces between octahedral cages and MA ions that result in different in-plane and out-of-plane tilting for various n values. The increasing n values introduce additional MA ions that increase the internal strain by steric effects, which will lead to the enhanced octahedral tilting degree. Therefore, n = 4 RPP has shortest I-I distance due to the largest our-of-plane tilt angles. In contrast, there is almost no out-of-plane tilt in n = 1 RPP so that it has the longest I-I distance. However, we found the out-of-plane octahedral tilt in even number of layers (n = 2 and n = 4) is larger than that in odd number of layers (n = 1 and n = 3) RPPs, which may due to the unbalanced internal charges in n = 2 (one-layer MA ion) and n = 4 RPPs (three layers MA ions). This result is also in agreement with former studies related to some inorganic perovskites. As a result of all these interactions, the out-of-plane tilt in n = 2 is larger than n

= 3 RPP, considering the syngeneic movement of in-plane tilt, the I-I distance in n = 2 is slightly shorter than that in n = 3 RPP.

3. The origins of the red emission in 2D perovskites remains to be under debate in the community. However, the most popular explanation is due to the edge state where the local structures at the edge facet should be different from the bulk structure. As the microscopic structure of the flake has already been provided in Fig. 5. I think the author should put more explanations to exclude other possible origins of the red emission, such as the edge emission or reabsorption of the emission light, etc.

ANS: According to reviewer's suggestion, we have closely examined the behaviour of edge PL emission by Micro-PL spectroscopy to exclude possible origins of the red emission as arising from edges.

(i) Ruling out the possibility of edge emission

As shown in Fig. R3, we have chosen 6 places on the exfoliated flake to check PL emissions (Fig. R3a). Locations 1,2,3 are at the interior region while locations 4,5,6 are at the edges. Fig. R3b and 3c show that the center and edge regions give the same dual PL emission (consisting of a main PL peak and a red-shifted peak). This indicates that the red-shifted emission does not arise solely from the crystal edge and is uniform across the sample. We should state that although edge states have been reported in 2D RPPs, they exhibit a broad PL emission at around 750 nm (***ACS Nano*** 2019, **13**, 2, 1635−1644; ***Science*** 2017, **355**, 1288–1292).

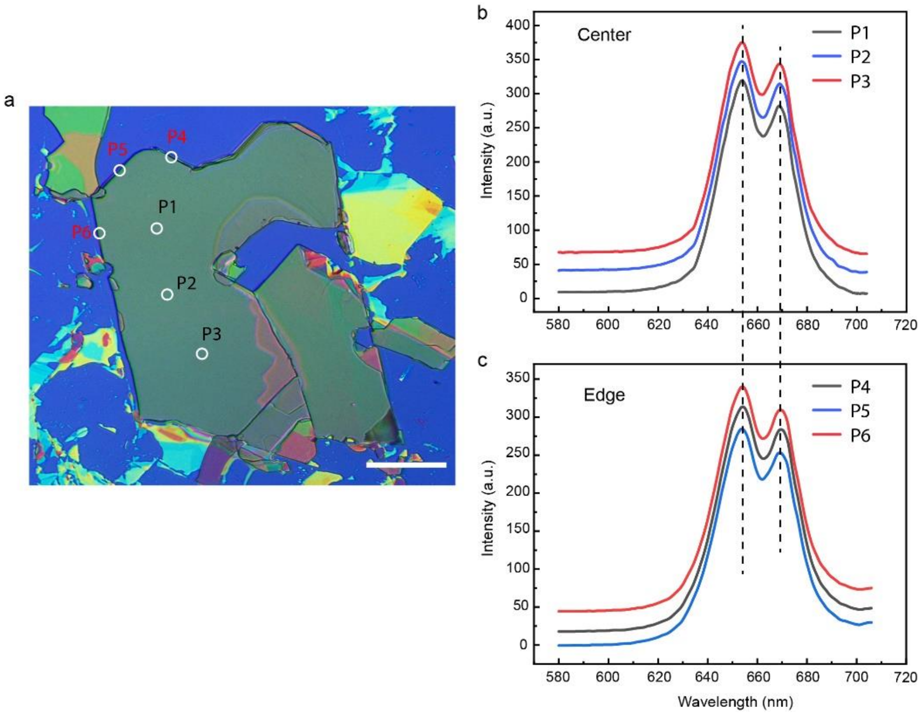

Fig. R3 | **a**, Optical image of exfoliated n = 4 RPP flake. Scale bar, 20 μm. **b**, Corresponding PL emissions of P1, P2 and P3 positions indicated in **a**. c, Corresponding PL emissions of P4, P5 and P6 positions indicated in **a.**

(ii) **Ruling out possibility of reabsorption**

The red-shifted peak observed in our case is quite sharp and is distinct from that causes by thickness-induced reabsorption shown in Fig R4. As shown in Fig. R4a, we demonstrate the "thickness induced reabsorption" by varying the thickness from bulk to monolayer, there is a discernible red-shifted component as thickness increases due to photon recycling effect. Similarly, as shown in Fig. R4b, Krahne *et al*. (***ACS Nano*** 2020, **14**, 4, 4689−4697) also observed a similar redshift of the emission peak with increasing thickness in $(PEA)_2PbBr_4$ flakes which is attributed to self-absorption. In both cases, we can see that the peak profile is very different from that formed after fresh exfoliation of the surface. **If the redshifted PL emission is caused by re-absorption, it should not be quenched by adding a layer of optically transparent h-BN layer on the perovskites surface. However, our result demonstrates that this red-shifted emission can be completely suppressed upon covering the surface of the exfoliated perovskite crystal with h-BN.**

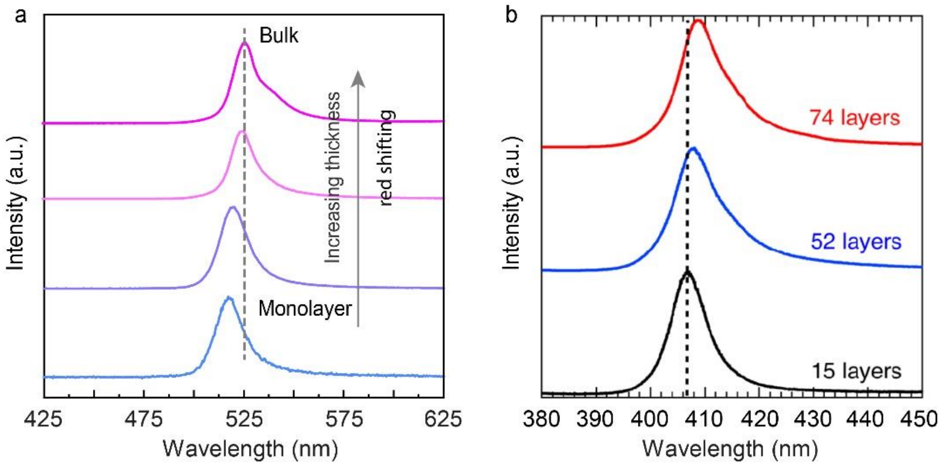

Fig. R4 | Photoluminescence (PL) spectra of RPPs with different thickness and the redshift of the PL peak with increasing thickness is due most likely to self-absorption. **a**, PL Red shift in Exfoliated $(BA)_2PbBr_4$ flakes (our result). **b**, PL Red shift in Exfoliated $(PEA)_2PbBr_4$ flakes (the result is cited from ***ACS Nano*** 2020, **14**, 4, 4689−4697. In the paper, author attributed this type of red shift to self-absorption).

We believe that the much sharper red-shifted PL peak observed here it is more likely due to surface reconstruction via BA-cation relaxation after exfoliating the surface, this relaxation extends across surface to the subsurface regions as discussed in our paper and gives rise to a redshifted PL. In addition, temperature-dependent PL measurements help to shed light on the origins of redshifted emission. If it is caused by reabsorption, the additional PL emissions should not show a big difference compared with main exciton as temperature decreases (***J. Mater. Chem. C***, 2020, **8**, 2289-2300). However, our redshifted emission decreases relative to the main exciton with temperature, due to decreased exciton-phonon coupling (Fig. 4d in manuscript and Fig. R13 below) as temperature decreases.

Action: We have added a related discussion in Page 9 of the revised manuscript as follows:

"Importantly, the red-shifted emission is uniform across the surface and is not related to edge emission[45, 46]. It also does not originate from thickness-dependent photon recycling because covering the surface with hexagonal boron nitride (h-BN) removes the red-shifted PL, independent of the flake thickness."

4. Would the applied bias during the STM measurement change the local structure specially the surface structure? Such possibility should also be excluded as it is important for the analysis of the surface lattice.

ANS: The changes are not due to ex-situ forces, but arises from intrinsic energy-dependent imaging of various orbitals that have different energy values with respect to the fermi level. As demonstrated in Fig. 2e and 2f in the manuscript, the topography of STM changed from dimer-like structure to quasi-square structure when the bias was changed from +2.3 V to +1.9 V in n = 4 RPP. However, the STM simulation (Supplementary Figure 7) clearly verified that this is due to the change of electron states contribution from apical I atoms to Pb atoms, excluding the possibility of real lattice changes with different applied bias.

Additionally, we have conducted bias-dependent STM on the same area of n = 4 RPP flake. As shown in the Fig. R5, the dimer-like structure is maintained when the bias decreases from +2.7 V to + 2.1 V (Fig. R5a-f), although the two atoms are becoming harder to distinguish at higher bias voltage due to the tip is going far away from the sample. For the bias below +2.0V (Fig. R5g and h), STM images show the same quasi-square structure that originate from the electron states of Pb atoms.

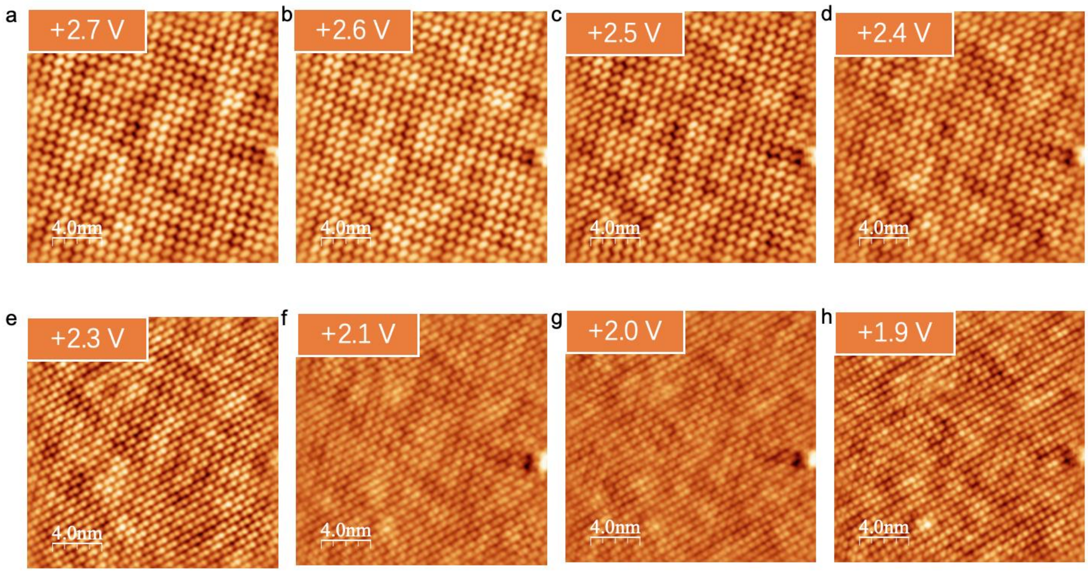

Fig. R5 | STM images of n = 4 RPP flake at various positive bias voltages.

We have also performed STM simulation under different bias voltages. As shown in the Fig. R6, at higher bias voltages, the image originates mainly from apical I atoms and the lattice remain the same with the bias voltage changes from +2.1 V to +2.7 V (Fig. R6a-c). At +1.9 V (Fig. R6d), the STM images changes because the signal is contributed by the Pb and in-plane I atoms underneath, not the structure change of apical atom. Therefore, we can

exclude the possibility of real lattice changes by various bias voltages during STM measurements.

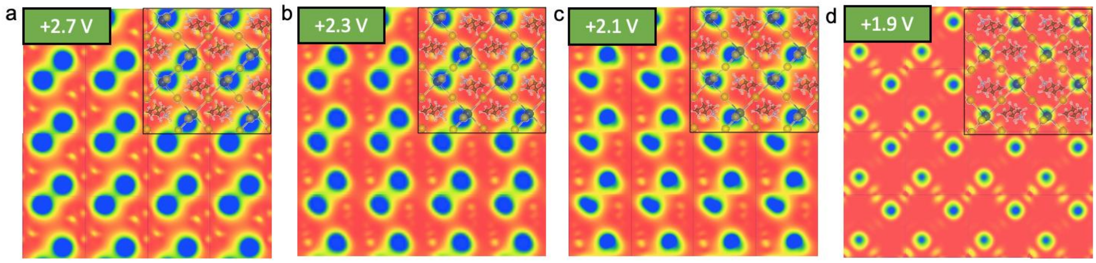

Fig. R6 | STM simulations of n = 4 RPP flake at various positive bias voltages.

Action: Following the reviewer's comments, we have added the bias-variable STM images and simulations to the revised supplementary information (as Supplementary Figure 7 and 8) to excluding the possibility of real lattice changes with different applied bias. We also added a sentence to clarify this in the revised manuscript at the end of Page 6 as follows:

"The influence of different applied bias on lattice structure during the STM measurement has been excluded by specific bias-variable STM measurements and corresponding STM simulations shown in the Supplementary Figure 7 and 8…… The different topologies of STM images at +1.9V and +2.3V are concluded to arise mainly from contributions from Pb and I orbitals, respectively."

Response to reviewer 2

Reviewer #2 (Remarks to the Author)

The manuscript describes a combined experimental and computational study of the tilting of octahedra in bulk and exfoliated quasi 2D perovskites. The main novelty is the characterization of the tilting of octahedra on the surface of the layers, which is different from the bulk. Attempts are made to correlate this with the observed optical properties. While the STM characterization of the tilting at the surface is new (as far as I know) I would rate the conceptual novelty of the presented work quite low. Distortions in perovskites (both 2D and 3D) are very well documented, both computationally and experimentally, primarily by X-ray. The latter means that there is mainly bulk information available and that the surface information obtained from STM is novel, but the differences observed are hardly surprising.

ANS: The reviewer has raised a good point. The previous studies were all concerned with BULK distortion, here we are looking at surface distortion, and we went to show that the surface induced octahedral tilts are larger than that of the bulk. Previous XRD studies cannot provide structural information that originates exclusively from the surface. We have searched the previous studies relating to the octahedral tilting (distortion) in both 2D and 3D perovskites and summarized their key findings and characterization techniques used to study the octahedral tilt in Table R1 and R2. As we can see, our work shows obvious differences compared with previous works. **1**. A large number of papers of octahedral tilt are reported in 3D perovskites induced by MA (***Chem. Mater.*** 2016, **28**, 12, 4259–4266). However, in 2D perovskite, the role of long organic space (BA) can not be neglected. In our paper, we emphasize after delaminating the perovskites and exposing the surface, it accentuates the surface octahedral tilt. **2**. Previous works reported that structural distortion result in broad or white light emission. In our work, the surface induced octahedral tilt result in sharp or narrow emission alongside the main exciton. **3**. Detailed structural information such as bond angles and bond lengths can only **be inferred indirectly from structure refinement of single crystal X-ray diffraction and DFT simulations**. **The direct experimental visualization of the microstructure of 2D perovskites at the atomic level is first reported by us**. Very recently, the atomic-resolution STEM imaging on 3D perovskite has just been reported in *Science* (***Science*** *2020,* ***370*** *(6516))*, this attests to the importance of atomic imaging.

It should be appreciated that it is highly challenging to perform STM on 2D perovskites due to the soft nature and complex species of organic/inorganic parts. **Our study provides the first atomic level real-space imaging of the surface structure from n = 1 to n = 4 hybrid 2D perovskites** and the direct evidence of the surface octahedral tilt and its correlation with the excitonic emission in photoluminescence.

On the other side, XRD studies are limited to **bulk averaged** information. The lack of microscope scale characterization limits the understanding of the property arise from the local structure in atomic level, such as from the surface and edges. For the first time, we focused on the unlocking of surface octahedral tilt upon exfoliation due to the unique soft

nature in 2D RPP interface, providing a unique view of surface enhanced inversion asymmetry in the 2D perovskites study.

Besides STM measurement, another sharp (narrow) emission alongside main exciton is first reported only in n > 1 2D RPPs by us.

In summary, we believe our findings can provide new insights for the 2D perovskite materials and the conception of our study is novel and inspiring.

Table R1 | Summary of former studies relating to structural distortions in **2D** hybrid perovskites.

Reference No.	Journal	Authors	Key findings	Techniques used to study structural distortion
1	ACS Nano	Matan et. al	Probe the evolution of the structural dynamics with temperature across the phase transition of $(BA)_2PbI_4$.	Polarization-orientation (PO) Raman scattering; DFT calculations;
2	Chem. Mater.	Stoumpos, et. al	Synthesis, crystal structure, and optical characterization of the 2D $(BA)_2(MA)_{n-1}PbnI_{3n+1}$ (n = 1,2,3,4)	Single-crystal X-ray diffraction; DFT calculations;
3	Adv. Electron. Mater.	Liang et. al	Reveal branched chain molecules aid to enhance the crystallization of 2D perovskites in comparison to linear chain counterparts	Single-crystal X-ray diffraction
4	Chem. Mater.	Legein et. al	Investigate the Hydrogen and Halogen Bonding interactions at the organic-inorganic interface which affect the distortion	Single-crystal X-ray diffraction; DFT calculations;
5	Dalton Trans.	Billing et. al	Crystal structure and phase transitions of $[(C_nH_{2n+1}NH_3)_2PbI_4]$, n = 7-10.	Single-crystal X-ray diffraction
6	Inorg. Chem.	Martin et. al	Tune the bandgap of 2d $(RNH_3)_2SnI_4$ by changing the Sn-I-Sn bond angle.	DFT calculations only
7	JACS	Kanatzidis et. al	Reveal the structural distortions can lead to a white-light emission in 2d $EA_4Pb_3Cl_{10}$	Powder and Single Crystal X-ray Diffraction.
8	JACS	Kanatzidis et. al	Synthesis, crystal structure, and optical and electronic properties of new type 2d DJ perovskites	Single-crystal X-ray diffraction
9	Angewandte Chemie	Luo et. al	Phase transition in 2d perovskite $(C_4H_9NH_3)_2(MA)_2Pb_3Br_{10}$ and reveal the ferroelectric effects.	Single-crystal X-ray diffraction

10	Chem. Mater.	Tisdale et.al	Synthesis, observed structural distortion and PL peak shifts across phase transitions in 2d perovskites.	Powder and Single Crystal X-ray Diffraction.
11	Chem. Mater.	Mohammed et. al	Layer-dependent Rashba effect in 2D perovskites which is associated with structural distortions.	DFT calculations only
12	Inorg. Chem.	Mitzi et. al	Reveal Pb−I−Pb bond angle between adjacent PbI_6 octahedra has a more global effect than other bond angles and lengths on the exciton properties	Single-crystal X-ray diffraction
13	JACS	Petrozza et. al	Reveal the structural distortions of the inorganic lattice play a key role in carrier localization and the subsequent broadband emission.	Single-crystal X-ray diffraction; Raman; DFT calculations;
14	Nat. Commun.	Mitzi et. al	The bond angle disparity connected with asymmetric tilting distortions breaks local inversion symmetry and strongly correlates with spin-splitting in 2d perovskites.	DFT calculations only

Table R2 | Summary of former studies relating to structural distortions in **3D** hybrid perovskites

Reference No.	Journal	Authors	Key findings	Techniques used to study structural distortion
1	Nat. Commun.	Giustino et.al	Octahedral tilting angles are strongly correlating with the optical band gap in 3d perovskites	DFT calculations only
2	Adv. Mater.	Xiao et. al	Observation of order-disorder transformation of MA cations which induced phase transitions and anomalous photoluminescence.	Single Crystal Neutron Diffraction Temperature-Dependent X-ray Diffraction
3	Chem. Comm.	Cheetham et. al	Reveal the strong hydrogen-bonding interaction with MA molecules and the tilting of the PbI_6 octahedra.	DFT calculations only
4	Chem. Comm.	Kieslich et. al	Investigate the variable temperature (100–450 K) and high pressure phase transition in	Single crystal X-ray diffraction; molecular dynamics

			$[(NH_2)_2CH]PbI$	(MD) simulations; DFT calculations
5	Chem. Mater.	Jang et. al	Reveal the hydrogen bonding between an organic A-cation and the halide frame plays a significant role in octahedral tilting of $MAPbI_3$ perovskite.	DFT calculations only
6	JACS	McGehee et. al	Identify two competing mechanisms through which A-site cation influences the band gap of 3D hybrid perovskites.	Single-crystal X-ray diffraction; DFT calculations;
7	Nano Lett.	Angelis et. al	Organic A-cation can tune the structure distortion and spin-obit coupling by the interplay of size effects and hydrogen bonding.	DFT calculations only
8	Nat. Mater.	Delaire et. al	Reveal structural instabilities and large atomic fluctuations that impact optical and thermal properties in $CsPbBr_3$	Single Crystal Neutron Diffraction; Single-crystal X-ray diffraction; DFT calculations;
9	PRL	Ross et. al	Reveal a general rule for predicting the variation in transition temperatures of tilt transitions in perovskite.	DFT calculations only

1. The connection that is made with the optical properties is quite weak: would DFT simulations (e.g., TDDFT) show the two different exciton peaks originating from different places in the film?

ANS: We thank the reviewer for this insightful comment. Following the reviewer's suggestion, we have performed additional DFT simulations to compare the electronic excitations between monolayer (exfoliated) and bulk RPP structure. We have simulated the case of n = 2 $(BA)_2(MA)Pb_2I_7$ perovskite which has the most prominent energy shift of the new emerging emission peak arisen from the enhanced surface octahedral tilt. Two methods including nonadiabatic molecular dynamic (NAMD) and time-dependent DFT (TDDFT) are applied for the DFT simulations.

Method 1: The nonadiabatic molecular dynamic based on PYXAID program (***J. Chem. Theory Comput.*** 2013, **9**,11, 4959–4972) is used to uncover the light emissions and reveal the dynamical structural distortions at room temperature. The calculation shows that, compared with the n = 2 bulk structure, the one unit of n = 2 RPP flake has additional absorption peaks, i.e., at ~635 nm (1.95 eV) and ~654 nm (1.90 eV) at the lower-energy side of the edge of main absorption (i.e., at ~594 nm (2.09 eV)) (Fig. R7a below). This clearly reveals that the structural distortion from the surface have significant effect on the light emission.

Method 2: Following the referee's suggestions, we have performed additional TDDFT simulation based on the *turbo_tddft* code (***Comput. Phys. Commun.*** 2014**,** ***185*** (7), 2080-2089) to compare the electronic excitations between monolayer (exfoliated) and bulk RPP structure. We have simulated the absorption spectrum for the case of *n* = 2 $(BA)_2(MA)Pb_2I_7$ perovskite who has the most prominent energy shift of the new emerging emission peak arisen from the enhanced surface octahedral tilt. The calculated TDDFT absorption spectrum (Fig. R7b below) clearly indicates an additional red-shifted peak appearing in monolayer n = 2 RPP at ~611 nm (2.03 eV), besides the main exciton peak existing in both bulk and monolayer at ~562 nm (2.20 eV). **This result clearly reveals that that surface octahedral tilt that is most pronounced in the monolayer has significant effect on the excitonic properties.**

It should be noted that the derived spectrum are obtained by simulation which is different from the experimental measured PL spectrum. Nevertheless, the TDDFT results indicate that there are excitonic excitations in the electronic band gap.

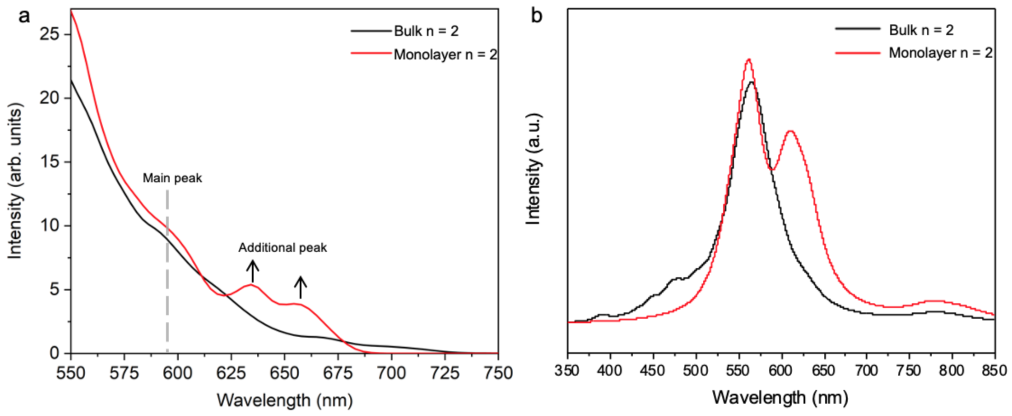

Fig. R7 | DFT simulated absorption spectra of both monolayer and bulk n = 2 RPP structure by **a**, Nonadiabatic molecular dynamic simulated method and **b**, Time-dependent DFT method. Both of the simulated optical absorption spectra show an additional emission in monolayer n = 2 RPP compared with its bulk.

Action: We have added the calculation result into the revised supplementary information (as Supplementary Figure 15) and a related discussion in Page 11 and Page 16 of the revised manuscript as follows:

"Our DFT simulation based on both nonadiabatic molecular dynamic and time-dependent DFT (TDDFT) methods corroborates the presence of a red-shifted exitonic peak in exfoliated n = 2 RPP in addition to the main exciton peak seen in the bulk crystal (Supplementary Fig. 15)."

"Nonadiabatic molecular dynamic and time-dependent DFT (TDDFT) methods suggest that as a result of the surface octahedral tilt, sub-gap excitonic states are created that can give rise to the redshifted PL."

2. It has been shown by confocal microscopy for exfoliated films that differences in emission are observed in the center of the flake and around the edges, is this the case for these fluorescence measurements?

ANS: Our confocal microscopy shows the same emission result whether the laser focused in the center or the edge. We have done the following experiment to address reviewer's concern.

As shown in Fig. R3 below, we have chosen 6 places on the exfoliated flake to check PL emissions (Fig. R3a). Locations 1,2,3 are at the interior region while locations 4,5,6 are at the edges. Fig. R3b and 3c show that the center and edge regions give the same dual PL emission (consisting of a main PL peak and a red-shifted peak). This indicates that the red-shifted emission does not arise solely from the crystal edge and is uniform across the sample. We should state that although edge states have been reported in 2D RPPs, they exhibit a broad PL emission at around 750 nm (***ACS Nano*** 2019, **13**, 2, 1635−1644).

Action: We have added a related discussion in Page 9 of the revised manuscript as follows:

"Importantly, the red-shifted emission is uniform across the surface and is not related to edge emission[45, 46]. It also does not originate from thickness-dependent photon recycling because covering the surface with hexagonal boron nitride (h-BN) removes the red-shifted PL, independent of the flake thickness."

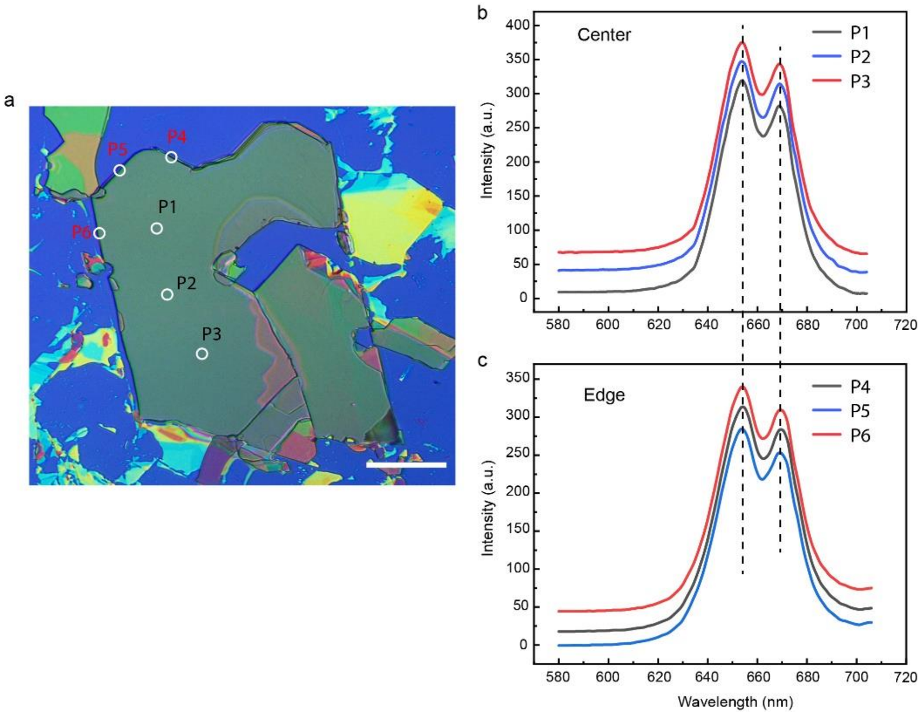

Fig. R3 | **a**, Optical image of exfoliated n = 4 RPP flake. Scale bar, 20 μm. **B**, Corresponding PL emissions of P1, P2 and P3 positions indicated in **a**. c, Corresponding PL emissions of P4, P5 and P6 positions indicated in **a.**

3. There is no information about the thickness of the flakes (number of monolayers). This can affect the optical and structural properties significantly. The same is true for the

interaction with the substrate, but in the calculations the substrate is not taken into account. The h-BN calculation show already the strong effect that can be expected here.

ANS: Thanks for this comment and we agree. As shown in Fig. R8a below, we have chosen an exfoliated n = 4 RPP flake with the similar optical contrast in manuscript to carry out AFM measurement. The redshifted emission alongside main exciton peak can be seen in this sample (Fig. R8b). Based on the height profile obtained by AFM (Fig. R8c), the corresponding thickness of the flake is about 150 nm. In this thickness range, the influence from the substrate can be neglected.

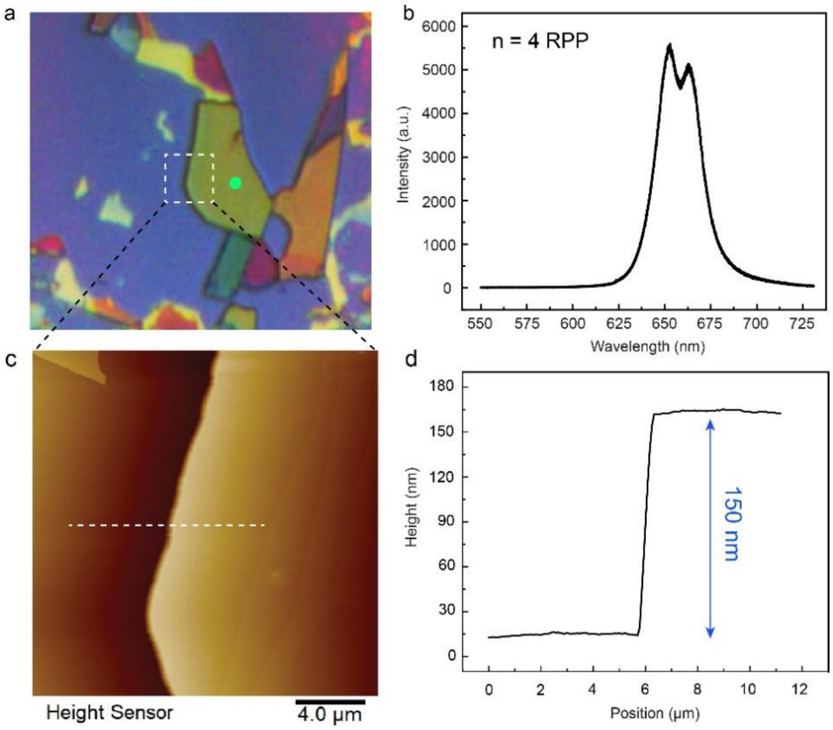

Fig. R8 | **a**, Optical image of exfoliated n = 4 RPP flake for AFM scanning. **b**, Corresponding PL emission from the position of green dot indicated in **a**. **c**, AFM image of the square region in **a**. **d**, Corresponding height profiles along the dotted line in **c**.

Action: we have added the thickness information in Page 9 of the revised manuscript as follows and added Fig. R8 (as Supplementary Figure 12) into the revised supplementary information

"However, the PL spectrum of exfoliated RPP flakes of ~100-150 nm thickness (Supplementary Fig. 12) reveal a sharp red-shifted emission peak in addition to the main exciton peak…"

Hexagonal boron nitride (h-BN) has a large band gap (~ 6 eV) and is optically transparent and inert. It is used as a top confinement layer on freshly exfoliated RPP with the purpose of investigating if it can suppress surface reconstruction and hence the redshifted emission. We have given a detailed discussion of the special role of h-BN in response of question 2 asked by reviewer #3.

Action: We have added a clearer description of special role of h-BN covering in Page 10 of revised manuscript for reviewer's concern.

" h-BN has a large band gap (~ 6 eV) and is optically transparent and inert. We used it as a top confinement layer on freshly exfoliated RPP with the purpose of investigating if it can suppress surface reconstruction and hence the redshifted emission."

4. Was the full exfoliation process done in inert atmosphere? Were the samples ever in contact with air during processing and measurements? In particular exfoliated BA-based layers are exceptionally prone to degradation.

ANS: The exfoliation was done in glove box in argon atmosphere away from water and oxygen. The samples were transferred in vacuum transfer box to a vacuum transfer station before introduction to UHV STM for performing STM. The very high atomic resolution obtained of the surface shows there is no sample degradation.

The PL studies also show no sample degradation as that will manifest as a broad red-shifted peak at about 700 nm (1.75 eV) regions (***Nat. Commun***. 2020, ***11*** (1), 2344), which was not observed. In fact, the FWHM of the PL peak of our samples are much sharper than that of previously reported samples.

5. The distinction between calculations and experimental results is not always clear and should be clearly indicate in the captions of figures.

Revision: We thank the reviewer for this comment. We have revised the captions of figures to clearly indicate the calculation and experimental results as follows:

(i) **Fig.1---** "Table 1, Comparison of the tilt angles of n = 4 RPP in monolayer surface and bulk interface according to DFT calculations."

(ii) **Fig.2---**"**e**, STM image scanned at V_{bias} = +2.3 V showing apical I atoms. I atom is indicated as bright yellow sphere in the simulation model. **f**, STM image scanned at V_{bias} = +1.9 V showing information of Pb atoms. Pb atom is indicated as black sphere in the simulation model. **g**, STM image scanned at V_{bias} = -2.3 V showing BA molecules in the simulation model."

(iii) **Fig.3---**"**a**,**b** Plot summarizing out-of-plane and in-plane octahedral tilt in the monolayer surface (yellow circles) and bulk interface (orange triangles) of n = 1, 2, 3, 4 RPP series according to the simulation models. The relative tilt (blue rectangles) is defined as the difference in tilt angle between monolayer and bulk octahedral tilt angle. **c**, The distance of two apical I atoms from the top of octahedrons according to the simulation models."

(iv) **Fig.5---**"**Table 2**, Comparison of the tilting angle of n = 2 RPP in non-exfoliated bulk interface, monolayer surface and h-BN covered monolayer surface according to the simulation models."

6. Given the abundance of X-ray data (and previous computational work) a comparison with that structural info would be in place.

ANS: The bulk structure of our RPP crystal is no different from the bulk structure of RPP crystals reported, and X-ray data we have already reported that in detail in the supporting information of *Leng et. al* (***Nat. Mater.*** *2018*, ***17***, *908*). The difference here is we are paying attention to surface structure of these crystal using STM which is the highlight of this work.

Is the referee requesting us to check how the surface structure differs from bulk structure determined by XRD? We compare the structure information of 2D RPPs obtained in our STM study with that from the single crystal X-ray diffraction study (***Chem. Mater.*** 2016, ***28*** (8), 2852-2867). The detailed comparisons of lattice distances of n > 1 RPPs are shown in Table R3-R5, the compared atom distances measured by STM and bulk XRD are marked in Fig. R9. Compared with structures from XRD data, the STM determined structure shows the shorter apical I-I distance (I_0-I_1) at the surface, indicating obvious octahedra tilting with higher degree of out-of-plane tilting. Lower crystal symmetry is found in our STM determined structure rather than that from XRD study.

Action: We have added the discussion of the structural comparison between our STM study with previous XRD data (in Supplementary Text) and corresponding figures and tables are put into the revised supplementary information as Supplementary Fig. 19 and Supplementary Table 9-11.

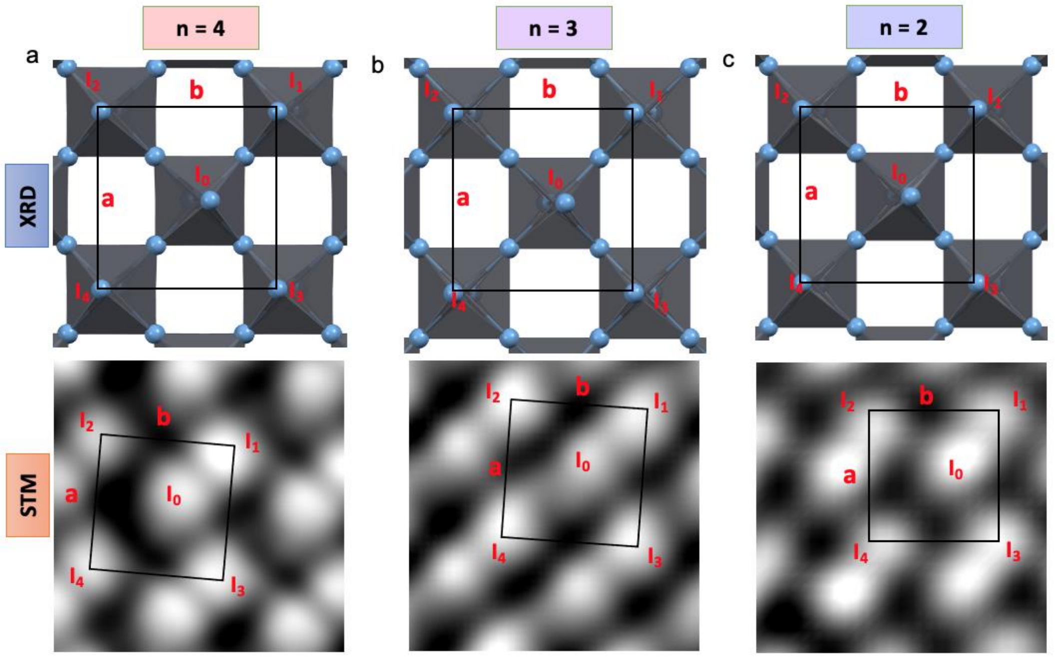

Figure R9 | Top-view illustration of n > 1 RPP structures from XRD (upper row) and STM (lower row) study, respectively. The unit cell is marked by the rectangle with lattice parameters a and b. I atoms are labeled for the comparison.

Table R3 | Comparison of surface structure of exfoliated n = 4 RPP by STM with previous reported structure by XRD, as indicated in Fig. R9a.

n = 4 RPP structure		STM on exfoliated RPP	XRD on bulk RPP
Atom distances (Å)	a	8.90	8.93
	b	8.51	8.88
	I_0-I_1	4.58	5.61
	I_0-I_2	6.87	7.07
	I_0-I_3	5.71	5.61
	I_0-I_4	7.12	7.07

Table R4 | Comparison of surface structure of exfoliated n = 3 RPP by STM with previous reported structure by XRD, as indicated in Fig. R9b.

n = 3 RPP structure		STM on exfoliated RPP	XRD on bulk RPP
Atom distances (Å)	a	9.13	8.93
	b	8.71	8.88
	I_0-I_1	5.02	5.59
	I_0-I_2	6.74	7.09
	I_0-I_3	6.34	5.59
	I_0-I_4	6.89	7.09

Table R5 | Comparison of surface structure of exfoliated n = 2 RPP by STM with bulk structure by XRD studies, as indicated in Fig. R9c.

n = 2 RPP structure		STM on exfoliated RPP	XRD on bulk RPP
Atom distances (Å)	a	8.80	8.95
	b	8.73	8.86
	I_0-I_1	4.85	5.59
	I_0-I_2	6.46	7.07
	I_0-I_3	5.42	5.59
	I_0-I_4	7.05	7.07

Response to reviewer 3

Reviewer #3 (Remarks to the Author)

The manuscript "Unlocking surface octahedral tilt in two-dimensional Ruddlesden-Popper perovskites" is very well written and structured, where they explained and justified the claims (i.e., octahedral tilt, formation of dimmers, etc) with solid proofs by authors. This is an exciting study, where they experimentally evidenced the octahedral tilts in RPPs (n = 1 to n = 4) and visualized it through STM. Also, the theoretical calculation supports the claimed result. It is interesting to see that, the different atoms can be visualized from the 2D-RPP through STM by different applied potentials. The layer dependent exciton-phonon study was also presented in a scientific manner, and the Rashba effect also supported them with DFT.

Therefore, this manuscript has recommended to accept after consideration of the fulfilment of the following major comments,

We thank the reviewer for the recognition of the solidity and great support to the manuscript. The point-by-point response to the reviewer #3's concerns are as follows:

1. Why does the in-plane octahedral tilt show less co-relation as compared to out of plane surface?

ANS: We attribute the red-shifted PL peak in the exfoliated 2D RPP flakes to the excitons coupling with phonons arising from the torsional motion of surface BA organic chains. Therefore, one explanation could be the fact that the out-of-plane tilt is a direct result of stronger vibronic coupling with the organic cations in that direction (***ACS Nano*** 2020, **14**, 4689−4697), this will cause a stronger exciton-phonon coupling leading to the redshift, comparing to the in-plane octahedral tilt.

Action: We have added this explanation into the revised manuscript in Page 13 as follows:

"One possible explanation is that the out-of-plane octahedral tilt results in a stronger vibronic coupling of the organic cations with the lead iodide octahedral cage compared to the in-plane octahedral tilt."

2. What is the significance of covering monolayers by h-BN? Why not other 2D materials? A minor discussion is lacking about the advantages of h-BN.

ANS: h-BN has a large band gap of 6 eV and is optically transparent and inert. It is commonly used as an inert passivating layer in 2D materials research. Upon splicing the interdigital organic bilayers in bulk crystal, only one layer of organic cations remain on the surface, and these will relax in response to reduced steric constraints (***Nat. Mater.*** 2018, **17**, 908–914) (Fig. R10a). The torsional motion of BA chains will couple to inorganic cages with interstitial MA and cause redshifted PL emission. In fact, Ryu *et al.*, once reported that surface reconstruction via MA cation ordering in $MAPbBr_3$, which extends from the crystal surface to the interior. The surface reconstruction can be suppressed upon surface treatment with PMMA coating as shown in Fig. R10b below(***J. Am. Chem. Soc.*** 2020, **142**, 21059−21067).

In this paper, we found that passivation by h-BN removes the red-shifted PL emission because h-BN suppresses the out-of-plane octahedral tilt (Fig. 5c in manuscript). We have also carried out DFT simulation by building model of monolayer RPP with a layer of h-BN stacked on top. Indeed, DFT calculations reveal that the out-of-plane relaxation is suppressed by covering with h-BN (Fig. 5e,f and Table 2 in manuscript).

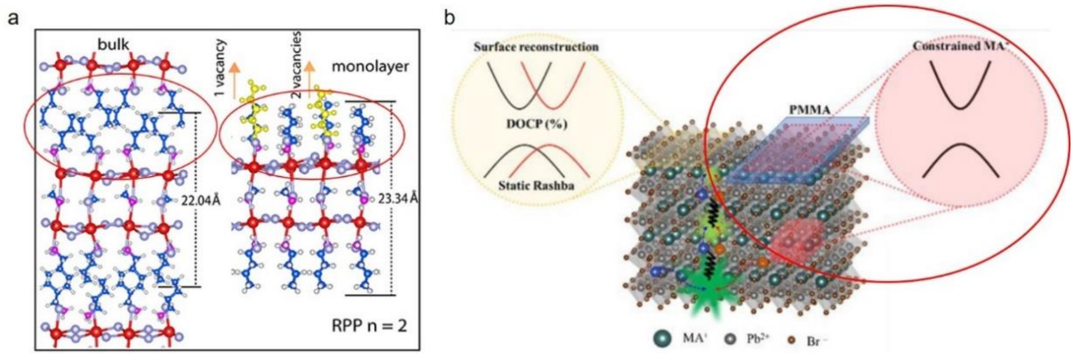

Fig. R10 | **a**, Schematic showing the relaxed RPP n = 2 monolayer versus bulk structure. **b**, Schematic showing static Rashba effect can be completely suppressed upon surface treatment with PMMA coating. Fig. R10a is cited from ***Nat. Mater.*** 2018, **17**, 908–914; Fig. R10b is cited from ***J. Am. Chem. Soc.*** 2020, **142**, 21059−21067.

Action: We have added the following description into Page 10 of the revised manuscript.

"h-BN has a large band gap (~ 6 eV) and is optically transparent and inert. We used it as a top confinement layer on freshly exfoliated RPP with the purpose of investigating if it can suppress surface reconstruction and hence the redshifted emission."

3. The fitting of figure 4d, could be much better than the existing one. Hence, the exponent may show different values.

ANS: In accordance with the reviewer's suggestion, we have re-fitted Fig. 4d in manuscript. As shown in Fig. R11 below, we varied the laser power by two orders of magnitude (similar with ***Phys. Rev. B*** 1992, **45** (16), 8989-8994). The new fitting results give k_{NE1} = 1.05 and k_{NE2} = 1.11 compared with previous k_{NE1} = 1.25 and k_{NE2} = 1.24 for main exciton and red-shifted exciton, respectively.

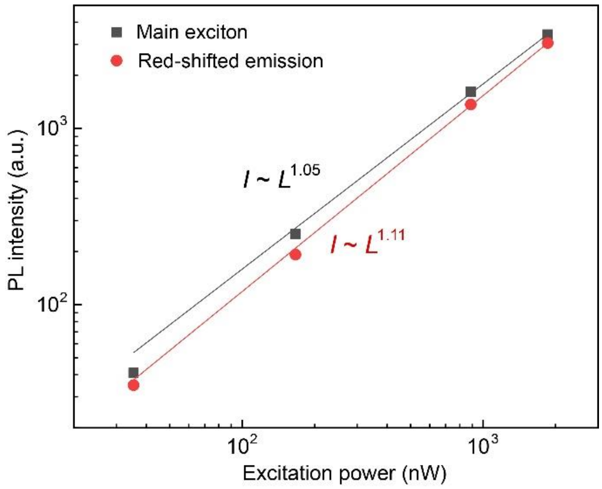

Fig. R11 | PL integrated intensity of the two excitonic emissions in n = 4 RPP, as a function of laser power.

Action: We have replaced the previous Fig. 4d in manuscript with above Fig. R11.

4. Exciton-phonon coupling has shown with PL, however, it can be shown more prominently with Raman spectroscopy, since the organic cation is polar. If possible, authors can perform the Raman spectra, and can involve these results in the main or supplementary manuscript.

ANS: Based on reviewer's suggestion, we have performed temperature-dependent Raman measurement on exfoliated flakes of n = 2 $(BA)_2(MA)Pb_2I_7$. The 633 nm Raman excitation is used to avoid excitation of strong PL background for n = 2 RPP. As shown in Fig. R12 below, three Raman peaks labelled as P1, P2 and P3 can be seen in the spectrum, corresponding to the vibrational modes of the organic cations of 2D RPPs.

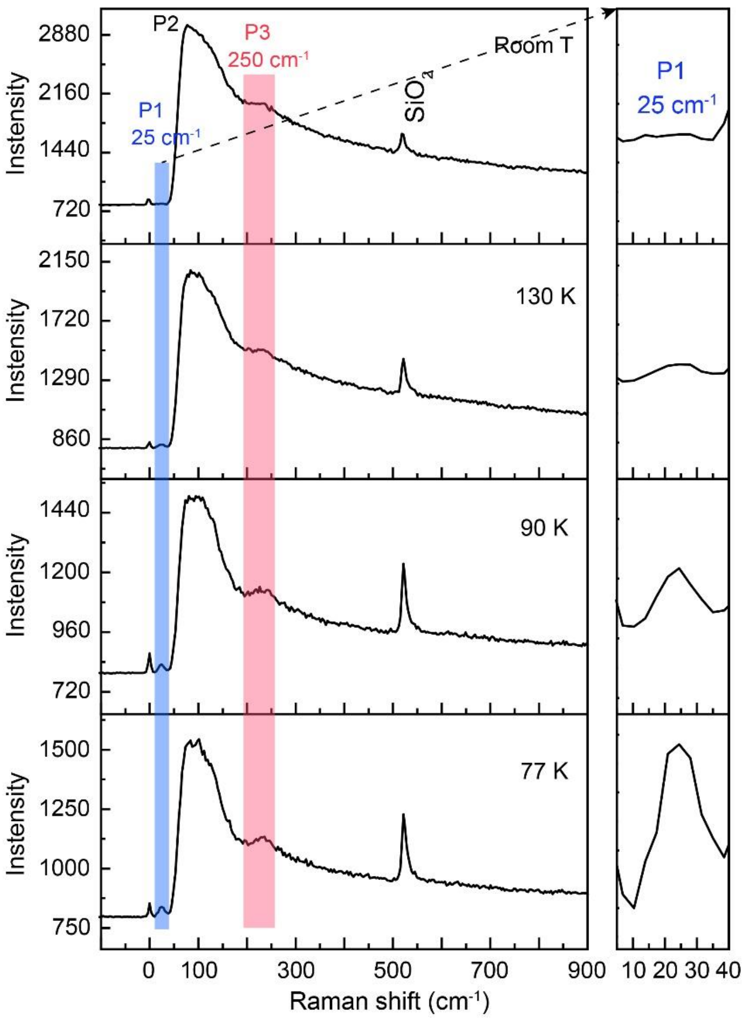

Fig. R12 | Temperature-dependent Raman spectra of exfoliated n = 2 $(BA)_2(MA)Pb_2I_7$.

As reported by a previous paper in ***J. Phys. Chem. Lett.*** 2020, **11**, 5830−5835, the rich, absorption spectra of $(PEA)_2(CH_3NH_3)_{n-1}Pb_nI_{3n+1}$ (where PEA stands for phenylethylammonium and n = 1, 2, 3), are related to a vibronic progression of excitonic

transition. In P3 band, the mode around 250 cm^{-1} is associated with the torsional mode of the NH_3^+ head of the organic spaces coupled to the exciton in $(BA)_2(MA)Pb_2I_7$ and this mode is sensitive to exciton-phonon coupling. The experimental data conclude that the enhancement of the Raman emission at low temperature results from: (i) an optical excitation near edge of fundamental absorption band; (ii) it is conditioned by the existence of excitonic PL. The authors point out the Raman peak around 240 cm^{-1} in $(BA)_2PbI_4$ is related to the coupled vibronic motion of $BANH_3^+$ with the octahedral cage. As shown, the 250 cm^{-1} band becomes increasingly sharp as temperatures decreases from room temperature to 77 K. In addition, in P1 range, the mode around 25 cm^{-1} is assigned to a twisting/rocking motion of the PbI_4 octahedra coupled to the vibration behaviour of organic moiety (***ACS Nano*** 2020, **14**, 4689−4697).

We have re-examined the temperature-dependent PL emission in exfoliated n = 4 sample. As show in Fig. R13, the red-shifted PL emission decreases in intensity with time as the temperature is lowered to 77 K, suggesting that it originates from exciton states that has coupled to phonon vibrations.

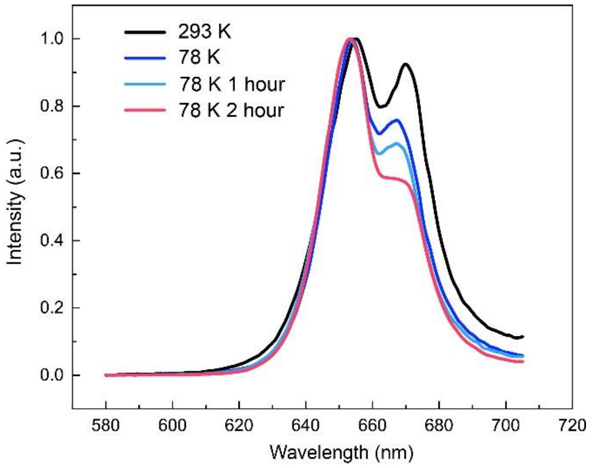

Fig. R13 | Temperature-dependent PL measurement in exfoliated n = 4 RPP.

Action: We have added Fig. R12 (as Supplementary Figure 20) and the corresponding discussion (in Supplementary Text) into the revised supplementary information as follows:

"Raman spectra measurement is carried out in exfoliated n = 2 RPP. In Supplementary Figure 20, the vibrational modes around 25 cm^{-1} and 250 cm^{-1} are associated with the torsional mode of the NH_3^+ head of the organic spaces that is coupled to excitonic transitions[3,4]."

5. Perovskite sample preparation for the STM measurement is quite tricky but crucial to obtain fine images. Could you mention the details of the sample preparations in the supplementary portion? So that majority of researchers can be benefitted and get enough idea of sample preparation of the kind of perovskite for SPM measurements.

Action: Following the reviewer's suggestion, we have added the detailed sample preparation process for the STM measurement into the revised supplementary information (as Supplementary Text and Supplementary Fig. 21) as follows:

"Single crystalline bulk 2D perovskite crystals with centimeter-size can be exfoliated into 2D thin films by mechanical-exfoliation method. The exfoliated flakes are then transferred onto Au or HOPG substrate for STM measurement. The exfoliation and transfer process were performed in an Ar-filled glove box. The sample is loaded in a vacuum transfer box into UHV chambers for STM. A long-focus camera is used to help locate the tip position. As illustrated in Fig. R14, RPP flakes with various thickness can be distinguished and measured by STM."

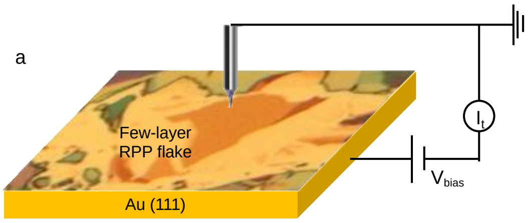

Figure R14 | Schematic illustration of the sample preparation for the STM measurement. **a**, Few-layer RPP flake for STM. **b**, Thick layer RPP flake for STM.

REVIEWERS' COMMENTS

Reviewer #1 (Remarks to the Author):

The authors have clearly stressed all my concerns and the revision greatly enhance the quality of the paper. Therefore I recommend publishing this paper in Nature Com. as it is.

Reviewer #2 (Remarks to the Author):

The authors have clarified many issues, raised by all reviewers and have added data that complements the original data, making the manuscript more complete. I feel that almost all issues raised are adequately addressed in the answer to comments of reviewers, but the changes in the manuscript are sometimes very minimal. The most important point is the justification of the study: in the response to comments of reviewer 2 the authors clearly outline the differences with previous studies and indicate the specific relevance of this work and it would help a lot if they find a way to also clarify this in the introduction of the paper, more than in the current version.

Reviewer #3 (Remarks to the Author):

The authors have fully addressed the questions raised by me and others. Therefore, I would like to suggest the acceptance of this manuscript for publication.

Point-by-point Response Letter

Reviewer #1 (Remarks to the Author):

The authors have clearly stressed all my concerns and the revision greatly enhance the quality of the paper. Therefore I recommend publishing this paper in Nature Com. as it is.

ANS: We thank the reviewer for this recommendation.

Reviewer #2 (Remarks to the Author):

The authors have clarified many issues, raised by all reviewers and have added data that complements the original data, making the manuscript more complete. I feel that almost all issues raised are adequately addressed in the answer to comments of reviewers, but the changes in the manuscript are sometimes very minimal. The most important point is the justification of the study: in the response to comments of reviewer 2 the authors clearly outline the differences with previous studies and indicate the specific relevance of this work and it would help a lot if they find a way to also clarify this in the introduction of the paper, more than in the current version.

ANS: Thanks for the suggestion. We have added the following sentences and revised the introduction part in Page 3 of the revised manuscript to justify our study.

"X-ray diffraction (XRD) had been widely used to investigate octahedral tilt or lattice distortion in both 2D and 3D perovskites (see the summary of relevant works listed in Supplementary Table 1 and Table 2). However, XRD provides bulk averaged information with no surface sensitivity, thus it cannot be used to probe the presence of surface reconstruction that occurs on exfoliated RPPs. Here, we directly imaged octahedral tilts on the surface of 2D RPPs $(BA)_2(MA)_{n-1}Pb_nI_{3n+1}$ (n = 1-4; BA: $CH_3(CH_2)_3NH_3$, n refers to the number of inorganic slabs per unit cell of the homologous series) using scanning tunnelling microscopy. We found that the octahedral tilt on the outermost layer of RPP was enhanced significantly compared to that of the bulk after "unlocking" the two interlocked layers of BA chains by mechanical exfoliation. In particular, the influence of MA ions on the octahedral tilt was investigated from $n = 1$ (without MA ions) to $n = 4$ ($n > 1$ has MA ions), from which we identified a clear trend between n and the strength of the octahedral tilt. Interestingly, the out-of-plane octahedral tilt of the exfoliated surface is correlated to the red-shifted emission peak alongside the primary exciton in the photoluminescence spectra, and also contributes to Rashba spin-splitting."

Reviewer #3 (Remarks to the Author):

The authors have fully addressed the questions raised by me and others. Therefore, I would like to suggest the acceptance of this manuscript for publication.

ANS: We thank the reviewer for this recommendation.
